# A New Compartmentalized Scale (*P*_N_) for Measuring Polarity Applied to Novel Ether-Functionalized Amino Acid Ionic Liquids

**DOI:** 10.3390/molecules27103231

**Published:** 2022-05-18

**Authors:** Xu Zheng, Chun Guo, Wenqing Wu, Jing Tong

**Affiliations:** College of Chemistry, Liaoning University, Shenyang 110036, China; zhengxu120512@163.com (X.Z.); guochun654321@163.com (C.G.); wwq18435221725@163.com (W.W.)

**Keywords:** polarity scale, ionic liquids, ether-functionalized, intermolecular interactions, molar surface entropy, Lorentz-Lorenz equation

## Abstract

Functionalized and environmentally friendly ionic liquids are required in many fields, but convenient methods for measuring their polarity are lacking. Two novel ether-functionalized amino acid ionic liquids, 1-(2-methoxyethyl)-3-methylimidazolium alanine ([C_1_OC_2_mim][Ala]) and 1-(2-ethoxyethyl)-3-methylimidazolium alanine ([C_2_OC_2_mim][Ala]), were synthesized by a neutralization method and their structures confirmed by NMR spectroscopy. Density, surface tension, and refractive index were determined using the standard addition method. The strength of intermolecular interactions within these ionic liquids was examined in terms of standard entropy, lattice energy, and association enthalpy. A new polarity scale, *P*_N_, is now proposed, which divides polarity into two compartments: the surface and the body of the liquid. Surface tension is predicted via an improved Lorentz-Lorenz equation, and molar surface entropy is used to determine the polarity of the surface. This new *P*_N_ scale is based on easily measured physicochemical parameters, is validated against alternative polarity scales, and is applicable to both ionic and molecular liquids.

## 1. Introduction

The green chemistry concept is a widely accepted focus of modern chemical research, including the design of new materials. Ionic liquids (ILs) have emerged as useful green reaction media due to their many unique features, such as low vapor pressure and high thermal stability [1,2]. They play important roles in many fields, including energy storage [3], catalysis [4], pharmaceuticals, and medicine [5,6]. However, the relatively high viscosity of ILs is a barrier to further practical applications. Inserting ether groups into the cations of ILs has been shown to substantially reduce their viscosity without lowering thermal stability, while also reducing their toxicity [7,8,9,10]. Ether-functionalized ILs (EFILs) have demonstrated remarkable performance in many fields. For example, they dissolve lignocellulosic biomasses [7], reduce viscosity and provide coordination sites for lithium ions in Li/Li-ion batteries [8], and enhance CO_2_ selectivity during CO_2_ capture [11]. However, traditional ILs containing anions such as Cl^−^, [BF_4_]^−^, and [PF_6_]^−^ are environmentally hazardous. The development of environmentally friendly, task-specific ILs based on renewable bioresources (such as amino acids and fatty acids) is an environmental necessity. Ohno and Fukumoto were the first to prepare amino acid ILs (AAILs), using 20 different amino acids as the anion; these AAILs demonstrated lower toxicity [12,13]. AAILs have now been utilized in enantioselective separation [14], extraction separation [15], and CO_2_ capture [11]. ILs with imidazolium-based cations exhibit low toxicity. Meanwhile, the shorter the alkyl chain on the imidazole ring, the lower the toxicity [16,17]. Thus, imidazolium ILs have proved more attractive than ILs based on ammonium, phosphonium, and pyridinium cations. The present study describes the preparation of two novel ether-functionalized, imidazolium-based AAILs: 1-(2-methoxyethyl)-3-methylimidazolium alanine ([C_1_OC_2_mim][Ala]) and 1-(2-ethoxyethyl)-face Tension, and Refractive Inde ([C_2_OC_2_mim][Ala]), together abbreviated as [C*_n_*OC_2_mim][Ala](*n* = 1, 2).

There is scope for considerable further research into EFILs because their physiochemical properties are highly susceptible to changes in structure. More experimental data are required to elucidate their structure–property relationships. This study measures the density, surface tension, and refractive index of [C*_n_*OC_2_mim][Ala](*n* = 1, 2). Density correlates with packing efficiency and intermolecular interactions and is a critical design property in chemical engineering [18]. Its study provides insight into the microstructure and macroscopic properties of ILs [19]. Surface tension is a crucial property at liquid–gas interfaces [20], affecting how the phases interact [21]. The surface tension of ILs is between those of alkanes and water [22]. It can be measured directly or predicted using, for example, the parachor formula [23] or group contribution methods [24]. This study predicts surface tension using an improved Lorentz-Lorenz equation. ILs are often used as solvents, so determining their polarity is crucial. Due to their non-structured nature, polarity cannot be determined by traditional methods such as relative permittivity (*ε*_r_) and dipole moment (*δ*) [25]. The most widely used experimental method for IL polarity is the *E*_T_(30) scale, which measures the solvatochromic UV−Vis absorbance shift of a solute. However, this method is time-consuming and expensive, so attempts have been made to develop predictive models [26,27].

The present study proposes a new polarity scale, *P*_N_, which enables polarity to be predicted from the easily measured physicochemical properties of density, surface tension, and refractive index. Following on from our previous studies [28,29,30], (i) [C*_n_*OC_2_mim][Ala](*n* = 1, 2) are synthesized and their structures confirmed by nuclear magnetic resonance spectroscopy (NMR); (ii) their density, surface tension, and refractive index are measured from 288.15 to 328.15 K at 5 K intervals; (iii) the strength of their molecular interactions are studied based on standard entropy, lattice energy, and association enthalpy; (iv) an improved Lorentz-Lorenz equation is used to predict the surface tension of ILs and molecular liquids; and (v) a new scale, *P*_N_, for estimating polarity is proposed, combining molar surface entropy *s* (which measures the polarity of the surface of a liquid) and the polarity coefficient *P*^2^ (which measures the polarity of the body of a liquid).

## 2. Results and Discussion

### 2.1. Density, Surface Tension, and Refractive Index of [C_n_OC_2_mim][Ala](n = 1, 2)

**Figure 1:**
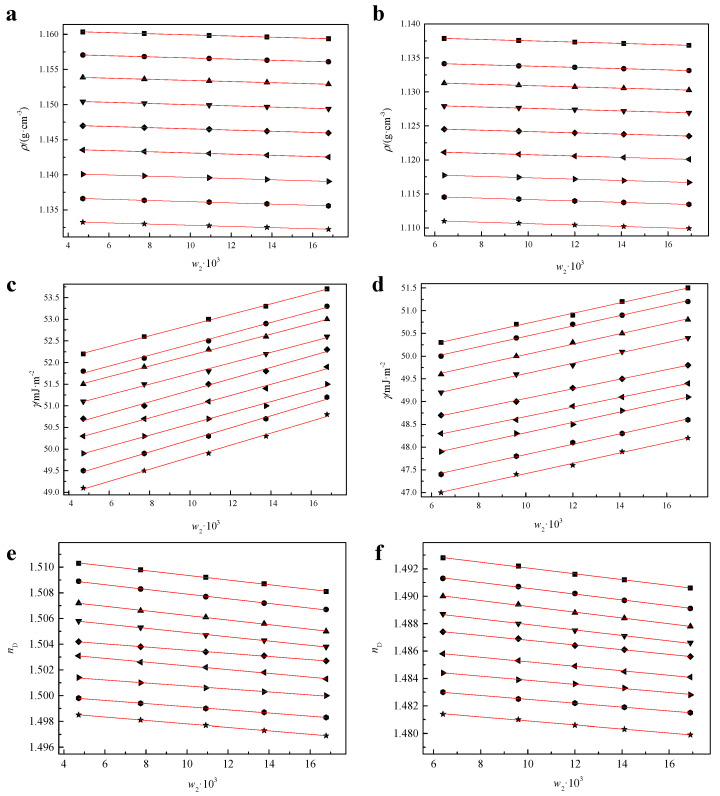
Density (*ρ*), surface tension (*γ*), and refractive index (*n*_D_) plotted against water content (*w*_2_) at various temperatures for [C_1_OC_2_mim][Ala] (**a**,**c**,**e**, respectively) and for [C_2_OC_2_mim][Ala] (**b**,**d**,**f**, respectively). ■ 288.15 K; ● 293.15 K; ▲ 298.15 K; ▼ 303.15 K; ◆ 308.15 K; ⏴ 313.15 K; ⏵ 318.15 K; ⯃ 323.15 K; ★ 328.15 K.

The density (*ρ*), surface tension (*γ*), and refractive index (*n*_D_) of [C*_n_*OC_2_mim][Ala](*n* = 1, 2) with various water contents over 288.15–328.15 K (at 5 K intervals) are shown in Tables S1–S3 Supplementary Materials, with each value being an average of three measurements using the standard addition method. These parameters were plotted against water content (Figure 1), producing a series of straight lines with correlation coefficient squares (*r*^2^) consistently greater than 0.99. The *y*-axis intercepts of these lines give the experimental value of each parameter in anhydrous [C*_n_*OC_2_mim][Ala](*n* = 1, 2) (Table 1).

### 2.2. Strength of [C_n_OC_2_mim][Ala](n = 1, 2) Intermolecular Interactions

The density of ILs increases gradually as temperature rises. At higher temperatures, the mobility of constituent ions improves and the unit volume increases [31]. The thermal expansion coefficient (*α*) is defined as

(1) $$\alpha =\left(1/V\right){\left(\partial V/\partial Y\right)}_{p}=-{\left(\partial \;ln\rho /\partial T\right)}_{p}$$

where *V* is molar volume. Molar volume is defined as

(2) V=M/ρ

The molecular volume *V*_m_ is defined as

(3) Vm=M/Nρ=V/N

where N is the Avogadro constant, *V* is molar volume, and M is molar mass.

At 298.15 K, *V*_m_ is 0.3300 nm^3^ for [C_1_OC_2_mim][Ala] and 0.3571 nm^3^ for [C_2_OC_2_mim][Ala] (Table S4). The difference between these indicates that the contribution of methylene (-CH_2_-) to *V*_m_ is 0.0271 nm^3^. This is close to the average contribution methylene makes to the *V*_m_ of several other ILs listed in Table S5 (0.0278 nm^3^).

Lattice energy (*U*_POT_) and standard entropy (*S*^θ^_298_) can be calculated according to Glasser’s theory; Equation (4) is suitable for MX(1:1) type ionic salts. The constants in Equation (5) are empirical values [32].

(4) $$U_{\mathrm{POT}}=1981.2\;{\left(\rho /M\right)}^{1/3}+103.8$$

(5) $${S^{\theta }}_{298}=1246.5Vm+29.5$$

*U*_POT_ reflects the strength of intermolecular interactions and can be used to measure the stability of ILs [32,33]. *U*_POT_ is 443 kJ·mol^−1^ for [C_1_OC_2_mim][Ala] and 435 kJ·mol^−1^ for [C_2_OC_2_mim][Ala], implying that methylene’s average contribution to *U*_POT_ is −8 kJ·mol^−1^. *U*_POT_ is inversely related to molar volume. Addition of a methylene group will reduce the ionic or molecular packing efficiency, decreasing the strength of the interactions.

To some extent, standard entropy reflects the degree of disorder of molecular arrangements [33]. *S*^θ^_298_ is 441 J·K^−1^·mol^−1^ for [C_1_OC_2_mim][Ala] and 475 J·K^−1^·mol^−1^ for [C_2_OC_2_mim][Ala], suggesting that methylene’s average contribution to *S*^θ^_298_ is 34 J·K^−1^·mol^−1^ (Table S5). This indicates that ILs with longer aliphatic chains are more disordered. Standard entropy increases as molecular volume increases. *S*^θ^_298_ of ILs is usually greater than 200 J·K^−1^·mol^−1^, compared with the more molecularly ordered conventional inorganic salts such as NaCl (72.1 J·K^−1^·mol^−1^) and KCl (82.6 J·K^−1^·mol^−1^) [34]. This may explain why ILs are molten below 373 K.

**Scheme 1:**
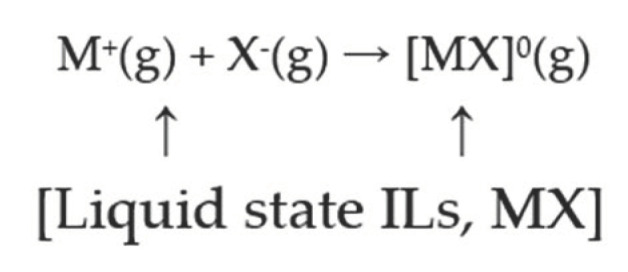
Thermodynamic cycle for calculating Δ_A_*H*_m_^0^.

The association enthalpy (Δ_A_*H*_m_^0^) also reflects the strength of intermolecular interactions: the higher the Δ_A_*H*_m_^0^, the stronger the gaseous state interactions. Δ_A_*H*_m_^0^ of ILs in the gaseous phase can be calculated based on the thermodynamic cycle shown in Scheme 1 [29].

Vaporization enthalpy (Δ_l_^g^*H*_m_^0^) is a key parameter for calculating Δ_A_*H*_m_^0^. It is estimated from Equation (6):

(6) $$g_{s}=a+b\;({\Delta_{l}}^{g}{H_{m}}^{0}\;-\;RT)$$

(7) $$g_{s}=\gamma V^{2/3}N^{1/3}$$

where *g*_s_ is the molar surface Gibbs energy; *γ* is surface tension; *V* is molar volume; N is the Avogadro constant; Δ_l_^g^*H*_m_^0^ is vaporization enthalpy; R is the gas constant; *T* is temperature; and a and b are the empirical constants −1.519 kJ·mol^−1^ and 0.09991, respectively [29].

The estimated vaporization enthalpy is 164.1 kJ·mol^−1^ for [C_1_OC_2_mim][Ala] and 165.1 kJ·mol^−1^ for [C_2_OC_2_mim][Ala]. Consequently, Δ_A_*H*_m_^0^ is −278.9 kJ·mol^−1^ for [C_1_OC_2_mim][Ala] and −269.9 kJ·mol^−1^ for [C_2_OC_2_mim][Ala]. The Δ_A_*H*_m_^0^ of other ILs are listed in Table S5. Again, addition of methylene reduces packing efficiency and increases the degree of molecular disorder. Thus, for ILs with the same anions, the absolute value of Δ_A_*H*_m_^0^ decreases as the length of the imidazole ring alkyl side chains increases. For ILs with the same cations, Δ_A_*H*_m_^0^ decreases with increasing anion volume.

### 2.3. Prediction of Surface Tension Based on Molar Surface Gibbs Energy

The parameter *g*_s_ used to estimate vaporization enthalpy was developed in our previous work by modifying Li’s model [35]. The definition of *g*_s_ is consistent with the concept presented by Myers [36]. Thus, *g*_s_ is a true thermodynamic function that integrates volumetric and surface properties.

**Figure 2:**
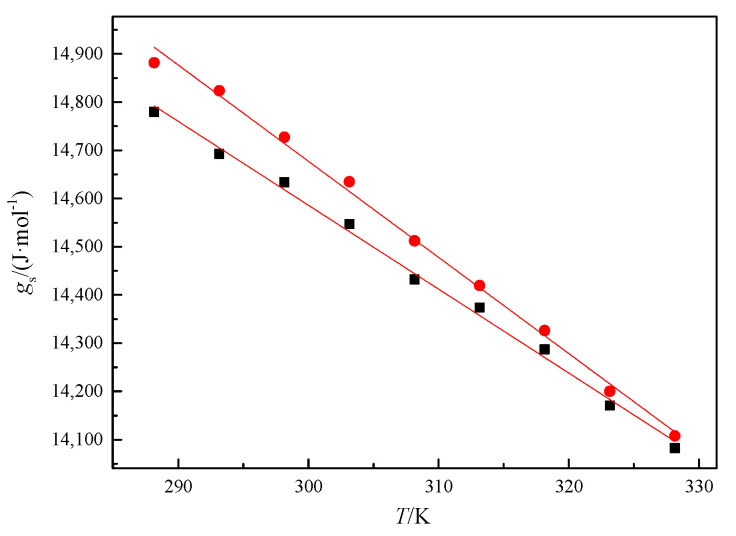
Molar surface Gibbs energy (*g*_s_) plotted against temperature (*T*). ■ [C_1_OC_2_mim][Ala]: *g*_s_ = 19,803 − 17.39*T*, *r*^2^ = 0.995, sd = 16.4; ● [C_2_OC_2_mim][Ala]: *g*_s_ = 20,658 − 19.94*T*, *r*^2^ = 0.996, sd = 17.5.

Plotting *g*_s_ against *T* for [C*_n_*OC_2_mim][Ala](*n* = 1, 2) yields straight lines (Figure 2) such that their relationship can be expressed as

(8) $$g_{s}=G_{0}\;-\;G_{1}T$$

*G*_0_ and *G*_1_ for [C*_n_*OC_2_mim][Ala](*n* = 1, 2) are obtained from Figure 2 and substituted into Equation (8) to give the estimated molar surface Gibbs energy, *g*_s(est)_. Values of *g*_s_, *G*_0_, *G*_1_, and *g*_s(est)_ are listed in Table 2.

The Lorentz-Lorenz equation expresses the relationship between *n*_D_ and the mean molecular polarizability (*α*_p_) [37]:

(9) $$R_{m}=[({n_{D}}^{2}\;-\;1)/({n_{D}}^{2}+2)]\cdot V=(4\pi N/3)\cdot \alpha_{p}$$

where *R*_m_ is molar refraction, *α*_p_ is mean molecular polarizability, and *n*_D_ is refractive index. This has been combined with *g*_s_ to give an improved Lorentz-Lorenz equation [38] that can predict surface tension, *γ*_(est)_:

(10) $${\gamma_{\left(\mathrm{est}\right)}}^{3/2}=[{g_{s(\mathrm{est})}}^{3/2}/N^{1/2}R_{m}]({n_{D}}^{2}-1)/({n_{D}}^{2}+2)$$

**Figure 3:**
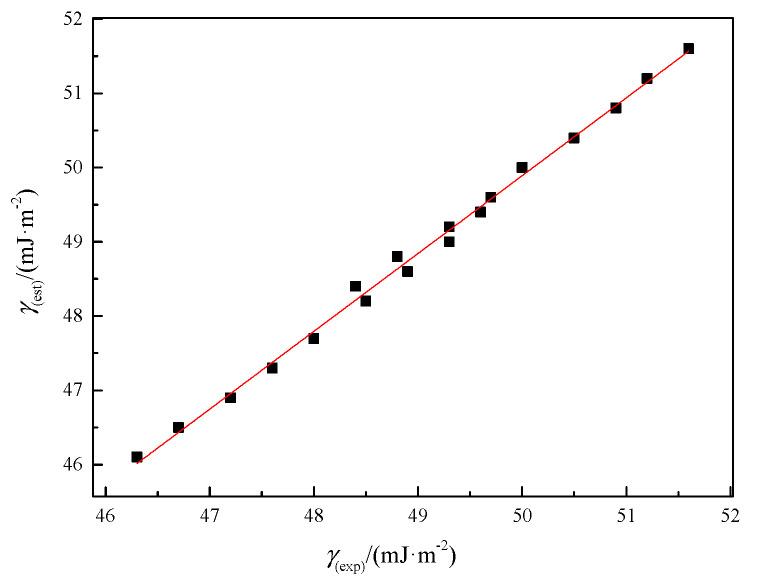
Plot of surface tension *γ*_(est)_ against *γ*_(exp)_ for [C*_n_*OC_2_mim][Ala](*n* = 1, 2). *γ*_(est)_ =1.0581*γ*_(exp)_ − 2.9851; *r*^2^ = 0.995; *sd* = 0.11.

**Figure 4:**
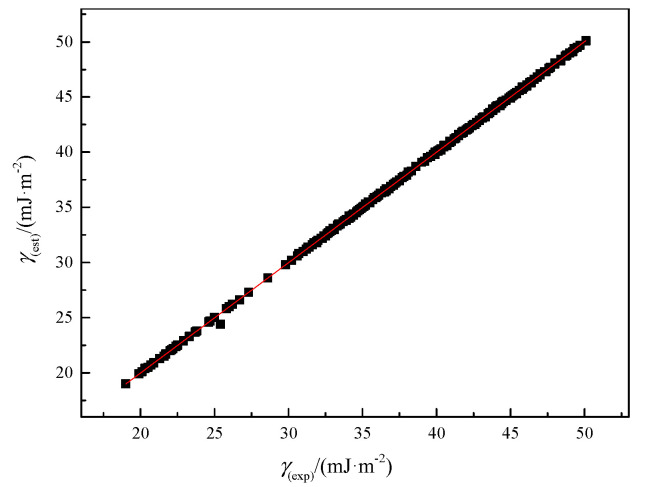
Plot of surface tension *γ*_(est)_ against *γ*_(exp)_ for various ionic and molecular liquids (see Table S6). *γ*_(est__)_ = 1.0009*γ*_(exp)_ − 0.03643; *r*^2^ = 0.999; *sd* = 0.10.

The *R*_m_, *α*_p_, and *γ*_(est)_ for [C*_n_*OC_2_mim][Ala](*n* = 1, 2) are listed in Table 3. Plotting estimated surface tension values against their corresponding experimental values produces a straight line (Figure 3). A similar plot for other ionic and molecular liquids also illustrates a linear relationship (Table S6, Figure 4), showing that this method is applicable for the surface tension prediction of both types of liquid.

For ILs sharing the same anions (Table 1 and Table S6), *n*_D_ decreases as the length of the alkyl chain in the cations increases. Refractive index correlates with dipole moment [39,40], which increases with higher molecular packing density [41]. Thus, a larger dipole moment results in a higher refractive index. The *n*_D_ is larger in [C_1_OC_2_mim][Ala] than [C_2_OC_2_mim][Ala], so the packing density of the former is higher, which is consistent with the density trend of the two ILs.

As polarizability increases, Coulomb interactions are reduced and ion mobility rises [42]. The *α*_p_ of [C_1_OC_2_mim][Ala] is lower than [C_2_OC_2_mim][Ala], so its Coulomb interactions are stronger. This finding is similar to the pattern observed for *U*_POT_ and Δ_A_*H*_m_^0^.

### 2.4. Molar Surface Entropy: Polarity Contribution from Surface Liquid

For most liquids, surface tension declines as temperature increases, as shown by the Eötvös equation:

(11) $$\gamma V^{2/3}=k(Tc\;-\;T)$$

where *T*_c_ is critical temperature and *k* is the Eötvös equation parameter, which is associated with polarity. For some organic liquids with weak polarity, *k* is nearly 2.2 × 10^−7^ J·mol^−2/3^·K^−1^, while for some with strong polarity, such as molten NaCl, it is nearly 0.4 × 10^−7^ J·mol^−2/3^·K^−1^ [35,43]. However, the physical significance of *k* is not clear. Multiplying both sides of the Eötvös equation by N^1/3^ [35] gives

(12) $$g_{s}=C_{0}\;-\;C_{1}T$$

which fits the fundamental thermodynamic concept:

(13) G=H−TS

*C*_1_ denotes the molar surface entropy and is given by *C*_1_= −($\frac{\partial g_{s}}{\partial T}$)_p_.

The relationship between molar surface entropy (defined here as *s* [29]) and *k* is expressed as

(14) $$s=N^{1/3}k$$

Entropy is directly linked to the number of microstates [44]. Higher entropy means molecules can be arranged in more ways, while the total energy remains constant. Thus, the physical significance of *s* is clear—it reflects the polarity of a liquid’s surface (higher *s*, lower surface polarity). The value of *s* is 17.39 J·mol^−1^·K^−1^ for [C_1_OC_2_mim][Ala] and 19.94 J·mol^−1^·K^−1^ for [C_2_OC_2_mim][Ala]. Values for other EFILs are listed in Table 4. The overall trend is that for ILs with the same cations, such as [C_1_OC_2_mim]^+^, [C_2_OC_2_mim]^+^, and [C_1_OC_4_mim]^+^, *s* increases as the volume of anions increases. Cl^−^, [Ala]^−^, [Thr]^−^, and [Gly]^−^ clearly obey this rule. It can be speculated that larger anions cause greater disordering of surface molecules, leading to lower polarity. However, [NTf_2_]^−^ does not conform to this rule. This may be due to it being more symmetrical than other anions, facilitating a more orderly arrangement of surface molecules and increasing the polarity. For ILs sharing the same anions, the general trend is that *s* increases as the volume of cations increases. Values of *s* for different other ILs (Table S7) confirm this effect.

Data for [C_1_OC_2_mim][NTf_2_], [C_2_OC_1_mim][NTf_2_], [C_2_OC_2_mim][NTf_2_], and [C_1_OC_3_mim][NTf_2_] (Table 4) imply that, for ILs with the same number of alkyl side chain carbons, the position of the ether group also affects *s*, presumably because it affects packing efficiency on the liquid surface.

### 2.5. A New Model for Predicting Polarity

Experimental methods for measuring polarity, such as *E*_T_(30), are time consuming and laborious. Here, we present a predictive model that establishes a relationship between polarity and the easily determined physicochemical properties of density, surface tension, and refractive index.

Our previous work [28], based on Hildebrand and Scott’s theory [47], proposed *δ*_μ_ as a polarity scale. *δ*_μ_ is the solubility parameter derived from the contribution of the average permanent dipole moment:

(15) $${\delta_{\mu }}^{2}={\Delta^{g}}_{l}{H^{0}}_{m\mu }/V\;-\;(1\;-\;x)\mathrm{RT}/V$$

where *V* is the molar volume and Δ^g^_l_*H*^0^_mμ_ is the contribution of the average permanent dipole moment to Δ^g^_l_*H*^0^_m_, such that

(16) $${\Delta^{g}}_{l}{H^{0}}_{m\mu }={\Delta^{g}}_{l}{H^{0}}_{m}\;-\;{\Delta^{g}}_{l}{H^{0}}_{\mathrm{mn}}$$

where Δ^g^_l_*H*^0^_mn_ is the contribution of the induced dipole moment to Δ^g^_l_*H*^0^_m_ and can be calculated from the Lawson–Ingham equation [48]:

(17) $${\Delta^{g}}_{l}{H^{0}}_{\mathrm{mn}}=C\;[({n_{D}}^{2}\;-\;1)\;/\;({n_{D}}^{2}+2)]V=C\;Rm$$

where C is the empirical constant 1.297 kJ·cm^−3^. In Equation (15), *x* represents Δ^g^_l_*H*^0^_mn_/Δ^g^_l_*H*^0^_m_ (at 298.15 K). The *δ_μ_* polarity of [C_1_OC_2_mim][Ala] (21.03 J^1/2^·cm^−3/2^) is larger than [C_2_OC_2_mim][Ala] (19.65 J^1/2^·cm^−3/2^).

However, there is an obvious drawback to *δ_μ_*: it has a dimension (J^1/2^·cm^−3/2^), while some polarity scales, such as the dielectric constant, are non-dimensional. Furthermore, the contribution of the induced dipole moment was neglected. Therefore, *δ_μ_* was improved as follows and designated as *P* [45]:

(18) $$P=\delta_{\mu }/\delta_{n}={\left[({\Delta^{g}}_{l}{H^{0}}_{m\mu }/V-(1-x)\;RT/V)/({\Delta^{g}}_{l}{H^{0}}_{\mathrm{mn}}/V-xRT/V)\right]}^{1/2}$$

where *δ*_n_ is the solubility parameter from the contribution of the induced dipole moment. Comparison of Δ^g^_l_*H*^0^_mμ_ and Δ^g^_l_*H*^0^_mn_ shows that (1−*x*)R*T*/*V* and *x*R*T*/*V* can be omitted and Equation (18) can be expressed as

(19) $$P={\left({\Delta^{g}}_{l}{H^{0}}_{m\mu }/{\Delta^{g}}_{l}{H^{0}}_{\mathrm{mn}}\right)}^{1/2}$$

The effects of average permanent dipole moment and induced dipole moment are both considered within *P*, which is dimensionless, with a large value indicating high polarity. [C_4_mim][BF_4_] is hydrophilic and [C_4_mim][NTf_2_] is hydrophobic. According to Seddon et al. [49], *P* is 1.226 for [C_4_mim][BF_4_] and 0.401 for [C_4_mim][NTf_2_]. This higher polarity of [C_4_mim][BF_4_] fits practical experience. Thus, the parameter *P* is capable of measuring the polarity of ILs. *P* is 1.191 for [C_1_OC_2_mim][Ala] and 1.043 for [C_2_OC_2_mim][Ala].

This study divided polarity into two compartments: the contribution from the body of a liquid, and the contribution from the surface. Cohesive energy density can demonstrate the strength of intermolecular interactions within the body [48]. *δ*_μ_^2^ is the cohesive energy density from the average permanent dipole moment and *δ*_n_^2^ is from the induced dipole moment. Consequently, *P*^2^ can describe the polarity of the body of a liquid:

(20) $$P^{2}={\delta_{\mu }}^{2}/{\delta_{n}}^{2}$$

Molar surface entropy (*s*) was proven above to reflect the polarity of a liquid surface. Combining *s* and *P*^2^, a new polarity scale, *P*_N_, is now proposed:

(21) $$P_{N}=s/P^{2}=s/({\delta_{\mu }}^{2}/{\delta_{n}}^{2})$$

This compartmentalized scale is a novel method to evaluate polarity, with a large *P*_N_ indicating weak polarity. Based on literature data [50,51,52,53,54,55], *P*_N_ is 22.03 J·mol^−1^·K^−1^ for [C_4_mim][NTf_2_] and 14.50 J·mol^−1^·K^−1^ for [C_4_mim][BF_4_]. These results fit with practical experience and demonstrate the rationality of the *P*_N_ scale. The *P*_N_ of [C_1_OC_2_mim][Ala] is 12.26 J·mol^−1^·K^−1^ and is 18.33 J·mol^−1^·K^−1^ for [C_2_OC_2_mim][Ala], which is the same polarity trend observed using *δ*_μ_ and *P*. As discussed above, ILs with longer alkyl chains exhibit higher standard entropy and lower molecular packing efficiency. The observed higher polarity of [C_1_OC_2_mim][Ala] may be due to its stronger intermolecular interactions and more ordered molecular arrangement. This fact can be explained as follows: a polar molecule has a permanent electric dipole moment, and a molecule may be polar if it has low symmetry [44]. ILs have asymmetric structures, and an orderly arrangement of ILs will maintain this structure. In this situation, their permanent electric dipole moments will not counteract each other, and polarity will be enhanced. The *P*_N_ of various ether-functionalized ILs are listed in Table 5. For ILs with the same anion, *P*_N_ declines as the length of the imidazole ring alkyl side chain increases. This trend supports the contention that the strength of intermolecular interactions and the degree of disorder of molecular arrangements influence polarity.

The *P*_N_ scale can be validated by comparison with other polarity scales (Table 6). FTIR spectroscopy probes [56] and *E*_T_^N^ [57] show that the polarity of [C_2_mim]BF_4_ is larger than [C_4_mim]BF_4_. The *P*_N_ scale gives the same qualitative result. Wu et al. determined the order of polarity of several ILs to be [C_4_mim]BF_4_ > [C_4_mim]NTf_2_ > [C_4_mim]OAc using *E*_T_^N^ [58]. Again, *P*_N_ gives the same result. 

**Figure 5:**
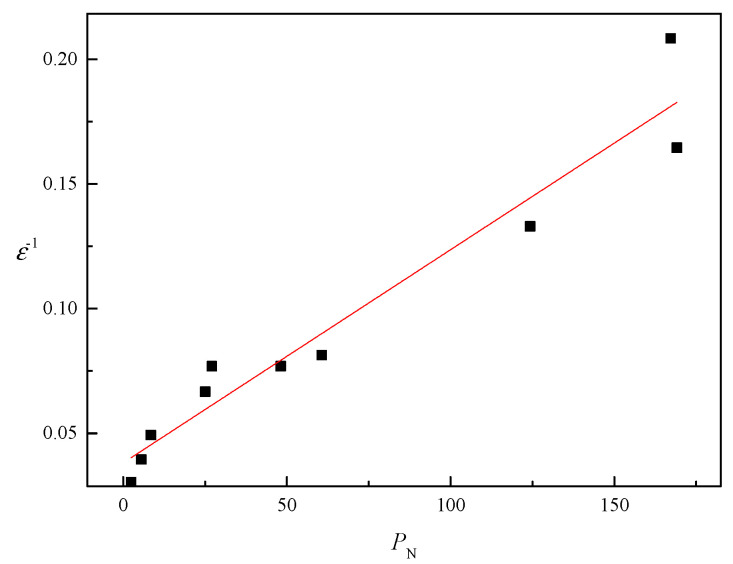
Inverse of the dielectric constant (*ε*^−1^) of various molecular liquids plotted against the estimated polarity *P*_N_. Linear correlation coefficient (*r*^2^) 0.94.

Furthermore, when *P*_N_ is applied to molecular liquids, the estimated polarity is broadly, and inversely, consistent with the dielectric constant *ε* [59] (Table 7). The correlation between *P*_N_ and the inverse *ε*^−1^ (Figure 5) is *r*^2^ = 0.94, demonstrating that *P*_N_ is also suitable for molecular liquids.

*E*_T_(30) is one of the most popular polarity scales for evaluating ILs, but it is laborious and costly [60]. Moreover, the results vary depending on the molecular probe used [61]. However, using *P*_N_, polarity can be determined simply from density, surface tension, and refractive index. The dielectric constant is the traditional polarity scale for organic solvents. The comparison of dielectric constant and *P*_N_ proves the universal applicability of *P*_N_. Thus, this new *P*_N_ scale is demonstrated to be a viable predictive method for evaluating the polarity of both ionic and molecular liquids based on easily measured physicochemical properties. It will find applications in many fields, particular those employing novel ionic liquids for which the evaluation of polarity is expensive and time consuming.

## 3. Materials and Methods

### 3.1. Materials

The sources and purity of reagents are listed in Table 8. *N*-Methylimidazole (AR grade) was purified by distillation, while 2-chloroethyl methyl ether and 2-chloroethyl ethyl ether (both AR grade) were used as purchased. DL-Alanine was recrystallized from water and dried in a vacuum oven [62].

### 3.2. Preparation of ILs [C_n_OC_2_mim][Ala](n = 1, 2)

**Figure 6:**
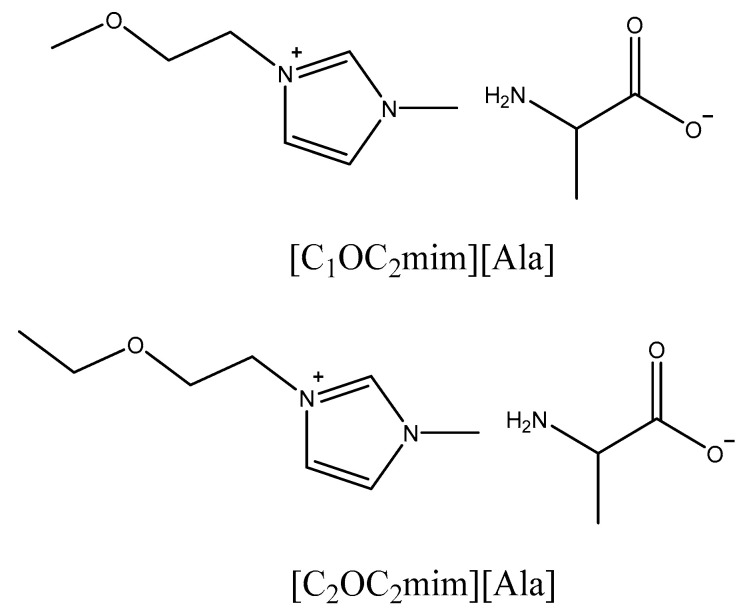
Chemical structures of ionic liquids [C*_n_*OC_2_mim][Ala](*n* = 1, 2).

[C*_n_*OC_2_mim][Ala](*n* = 1, 2) were prepared by Fukumoto’s neutralization method [12], and [C*_n_*OC_2_mim]Cl(*n* = 1, 2) by Sheldon’s method [63]. An equal molar amount of 2-chloroethyl methyl ether or 2-chloroethyl ethyl ether was added dropwise to *N*-methylimidazole under nitrogen in a three-necked round-bottom flask while stirring at 298.15 K. The reaction temperature reached 353.15 K with 2-chloroethyl methyl ether and 373.15 K with 2-chloroethyl ethyl ether. The reactions lasted for 48 h and produced light yellow liquids, which were then washed three times with ethyl acetate, producing [C*_n_*OC_2_mim]Cl(*n* = 1, 2). These were transformed into [C*_n_*OC_2_mim]OH(*n* = 1, 2) using basic anion exchange resin conditioned with sodium hydroxide [29]. The aqueous [C*_n_*OC_2_mim]OH(*n* = 1, 2) were then added dropwise (at slight excess) to aqueous DL-alanine and reacted for 72 h, yielding [C*_n_*OC_2_mim][Ala](*n* = 1, 2). Water was removed by rotary evaporation and excess DL-alanine by ethanol:acetonitrile (9:1). The solvents were evaporated under reduced pressure and [C*_n_*OC_2_mim][Ala](*n* = 1, 2) were dried in a vacuum oven for 60 h at 353.15 K. The chemical structures of [C*_n_*OC_2_mim][Ala](*n* = 1, 2) are shown in Figure 6.

### 3.3. Analytical Methods

The structures of [C*_n_*OC_2_mim][Ala](*n* = 1, 2) were characterized by NMR (Varian XL-300), as shown in the Supplementary Materials. The final water contents (*w*_2_) of [C*_n_*OC_2_mim][Ala](*n* = 1, 2), as measured using a ZSD-2 Karl Fischer moisture titrator, were 0.00472 and 0.00640 ± 0.0001 (mass fraction), respectively. 

Since [C*_n_*OC_2_mim][Ala](*n* = 1, 2) form strong hydrogen bonds with water, it is difficult to remove all traces of water from these ILs, which affects their density, surface tension, and refractive index. Therefore, the standard addition method was used to determine these properties. Each parameter was measured in [C*_n_*OC_2_mim][Ala](*n* = 1, 2) at different water contents following heating at graduated temperatures. Values were then plotted against water content, and the intercept of the regression lines yielded the parameter values in the anhydrous ILs at a given temperature.

## 4. Conclusions

[C*_n_*OC_2_mim][Ala](*n* = 1, 2) were prepared and their structures confirmed by NMR. Density, surface tension, and refractive index were determined by the standard addition method. Adding methylene to the aliphatic chain of an IL increased its standard entropy. Lattice energy and association enthalpy measurements showed that molecules of [C_1_OC_2_mim][Ala] were more compacted, and their intermolecular interactions stronger, than [C_2_OC_2_mim][Ala]. An improved Lorentz-Lorenz equation predicted the surface tension of both ionic and molecular liquids. A new compartmentalized polarity scale (*P*_N_) based on molar surface Gibbs energy and dipole moments is presented. It encompasses the polarity of both the surface and body of a liquid. [C_1_OC_2_mim][Ala] is shown to have higher polarity than [C_2_OC_2_mim][Ala] based on *P*_N_. The validity of *P*_N_ is demonstrated by comparison with alternative polarity scales and published polarities of both ionic and molecular liquids.

## Figures and Tables

**Table 1:** Density (*ρ*), surface tension (*γ*), refractive index (*n*_D_), and thermal expansion coefficient (*α*) at various temperatures (*T*) for [C*_n_*OC_2_mim][Ala](*n* = 1, 2).

*T* (K)	*ρ* (g·cm^−3^)	*γ* (mJ·m^−2^)	*n*_D_	*α* (K^−1^ × 10^4^)
[C_1_OC_2_mim][Ala]				
288.15	1.16073	51.6	1.5112	5.8555
293.15	1.15744	51.2	1.5097	5.8722
298.15	1.15423	50.9	1.5080	5.8885
303.15	1.15082	50.5	1.5066	5.9060
308.15	1.14739	50.0	1.5048	5.9236
313.15	1.14395	49.7	1.5038	5.9414
318.15	1.14049	49.3	1.5019	5.9595
323.15	1.13703	48.8	1.5004	5.9776
328.15	1.13365	48.4	1.4991	5.9954
[C_2_OC_2_mim][Ala]				
288.15	1.13849	49.6	1.4942	5.8490
293.15	1.13475	49.3	1.4927	5.8683
298.15	1.13190	48.9	1.4914	5.8830
303.15	1.12854	48.5	1.4899	5.9005
308.15	1.12514	48.0	1.4885	5.9184
313.15	1.12175	47.6	1.4869	5.9363
318.15	1.11838	47.2	1.4854	5.9541
323.15	1.11521	46.7	1.4839	5.9711
328.15	1.11166	46.3	1.4824	5.9901

Standard uncertainties (*u*) are *u*(*T*) = 0.02 K and *u*(*p*) = 10 kPa; expanded uncertainties (*U*) are *U*(*ρ*) = 0.002 g·cm^−3^, *U*(*γ*) = 0.3 mJ·m^−2^, and *U*(*n*_D_) = 0.003, with 95% confidence (*k* = 2).

**Table 2:** Molar surface Gibbs energy (*g*_s_), *G*_0_, *G*_1_, and estimated molar surface Gibbs energy (*g*_s(est)_) for [C*_n_*OC_2_mim][Ala](*n* = 1, 2).

*T* (K)	*g*_s_ (kJ·mol^−1^)	*G*_0_	*G*_1_	*g*_s(est)_ (kJ·mol^−1^)
[C_1_OC_2_mim][Ala]				
288.15	14.78	19,803	17.4	14.79
293.15	14.69	19,803	17.4	14.70
298.15	14.63	19,803	17.4	14.62
303.15	14.55	19,803	17.4	14.53
308.15	14.43	19,803	17.4	14.44
313.15	14.37	19,803	17.4	14.35
318.15	14.29	19,803	17.4	14.27
323.15	14.17	19,803	17.4	14.18
328.15	14.08	19,803	17.4	14.09
[C_2_OC_2_mim][Ala]				
288.15	14.88	20,658	19.9	14.92
293.15	14.82	20,658	19.9	14.82
298.15	14.73	20,658	19.9	14.72
303.15	14.63	20,658	19.9	14.63
308.15	14.51	20,658	19.9	14.53
313.15	14.42	20,658	19.9	14.43
318.15	14.33	20,658	19.9	14.33
323.15	14.20	20,658	19.9	14.23
328.15	14.11	20,658	19.9	14.13

**Table 3:** Molar refraction (*R*_m_), mean molecular polarizability (*α*_p_), and estimated surface tension (*γ*_(est)_) of [C*_n_*OC_2_mim][Ala](*n* = 1, 2).

*T* (K)	*R*m	*α*_p_ × 10^24^	*γ*_(est)_
[C_1_OC_2_mim][Ala]			
288.15	57.74	22.91	51.6
293.15	57.77	22.92	51.2
298.15	57.76	22.92	50.8
303.15	57.80	22.93	50.4
308.15	57.82	22.94	50.0
313.15	57.84	22.95	49.6
318.15	57.89	22.97	49.2
323.15	57.90	22.97	48.8
328.15	57.89	22.97	48.4
[C_2_OC_2_mim][Ala]			
288.15	62.24	24.69	49.4
293.15	62.28	24.71	49.0
298.15	62.30	24.72	48.6
303.15	62.32	24.73	48.2
308.15	62.36	24.74	47.7
313.15	62.37	24.75	47.3
318.15	62.39	24.76	46.9
323.15	62.41	24.76	46.5
328.15	62.43	24.77	46.1

**Table 4:** Molar surface entropy (*s*) of various EFILs.

IL	*s* (J·mol^−1^·K^−1^)	V × 10^4^ (m^3^·mol^−1^)
[C_1_OC_2_mim]Cl [38]	15.99	1.52
[C_1_OC_2_mim][Ala]	17.39	1.99
[C_1_OC_2_mim][Thr] [45]	26.60	2.18
[C_1_OC_2_mim][NTf_2_] [19]	17.80	2.80
[C_2_OC_2_mim]Cl [38]	17.81	1.68
[C_2_OC_2_mim][Ala]	19.94	2.15
[C_2_OC_2_mim][Thr] [45]	28.12	2.37
[C_2_OC_2_mim][NTf_2_] [19]	19.32	2.99
[C_1_OC_4_mim][Gly] [46]	19.56	2.19
[C_1_OC_4_mim][Ala] [46]	20.54	2.29
[C_1_OC_4_mim][Thr] [46]	22.082	2.51
[C_2_OC_1_mim][NTf_2_] [19]	18.20	2.81
[C_1_OC_3_mim][NTf_2_] [19]	18.49	2.97
[C_3_OC_2_mim][NTf_2_] [19]	20.57	3.16

**Table 5:** Polarity of various ether-functionalized ionic liquids using the new *P*_N_ scale.

IL	*P*_N_ (J·mol^−1^·K^−1^)	Reference
[COC_2_mim]Cl	12.86	[37]
[C_2_OC_2_mim]Cl	16.76	[37]
[C_1_OC_2_mim][Ala]	12.26	This work
[C_2_OC_2_mim][Ala]	18.33	This work
[COC_4_mim][Ala]	21.83	[39]
[COC_2_mim][Thr]	22.18	[38]
[C_2_OC_2_mim][Thr]	25.46	[38]
[COC_4_mim][Thr]	24.52	[39]
[COC_2_mim][NTf_2_]	31.31	[13]
[C_2_OCmim][NTf_2_]	35.40	[13]
[C_1_OC_3_mim][NTf_2_]	35.57	[13]
[C_2_OC_2_mim][NTf_2_]	42.66	[13]
[C_3_OC_2_mim][NTf_2_]	55.1	[13]
[COC_4_mim][Gly]	19.64	[39]

**Table 6:** Polarity of various ionic liquids estimated using the *P*_N_ scale.

IL	*P*_N_ (J·mol^−1^·K^−1^)
[C_6_mim]OAC	35.73
[C_4_mim]OAC	23.24
[C_4_mim]NTf_2_ [51,53]	22.03
[C_4_mmim]NTf_2_	19.96
[C_4_mim]BF_4_	14.50
[C_2_mmim]NTf_2_	14.01
[C_5_mim]Lact	12.87
[C_2_mim]BF_4_	8.24
[C_2_mim]Lact	8.16

**Table 7:** *P*_N_ and dielectric constant (*ε*) of various molecular liquids.

Molecular Liquid	*P*_N_	*ε*
Ethyl acetate	169.14	6.1
Chloroform	167.19	4.8
Tetrahydrofuran	124.29	7.5
Pyridine	60.64	12.3
Benzyl alcohol	48.13	13.0
1-Hexanol	27.06	13.0
Cyclohexanol	25.09	15.0
1-Propanol	8.48	20.3
Ethanol	5.52	25.3
Methanol	2.40	33.0

**Table 8:** Source and purity of reagents.

Reagent Name	CAS No.	Source	Purification	Mass Fraction Purity	Analysis
Anion exchange resin 717	122560-63-8	SRC	None	Granularity > 0.950	GC
*N*-Methylimidazole	616-47-7	ACROS	Distillation	>0.990	FM
2-Chloroethyl methyl ether	627-42-9	SRC	None	>0.995	FM
2-Chloroethyl ethyl ether	628-34-2	SRC	None	>0.995	FM
DL-Alanine	302-72-7	SRC	Recrystallization	>0.990	FM
Acetonitrile	75-05-8	SRC	None	>0.995	FM
Ethyl acetate	141-78-6	SRC	None	>0.995	FM
Anhydrous ethanol	64-17-5	SRC	None	>0.995	FM
Sodium hydroxide	1310-73-2	SRC	None	>0.960	FM
[C_1_OC_2_mim][Ala]	-	Synthesis	Solvent extraction, vacuum drying	>0.990	^1^H, ^13^C NMR
[C_2_OC_2_mim][Ala]	-	Synthesis	Solvent extraction, vacuum drying	>0.990	^1^H, ^13^C NMR

FM—Fractional melting; SRC—Shanghai Reagent Co., Ltd.

## Data Availability

Not applicable.

## Supplementary Materials

The following supporting information can be downloaded at: https://www.mdpi.com/article/10.3390/molecules27103231/s1, Figure S1. ^1^H NMR spectrum of IL [COC_2_mim][Ala]; Figure S2. ^1^H NMR spectrum of IL [C_2_OC_2_mim][Ala]; Figure S3. ^13^C NMR spectrum of IL [COC_2_mim][Ala]; Figure S4. ^13^C NMR spectrum of IL [C_2_OC_2_mim][Ala]; Table S1. Densities of Ionic Liquids Containing Various Amounts of Water at pressure *p* = 0.1 MPa; Table S2. Surface Tensions of Ionic Liquids Containing Various Amounts of Water at pressure *p* = 0.1 MPa; Table S3. Refractive Indexes of Ionic Liquids Containing Various Amount of Water at pressure *p* = 0.1 MPa; Table S4. The values of molar volume, *V*/cm^3^·mol^−1^ and molecular volume, *V*_m_/nm^3^ for the ILs [C*_n_*OC_2_mim][Ala](*n* = 1, 2); Table S5. Molecular volume, *V*_m_, standard molar entropy, *S*^0^, lattice energy, *U*_POT_, vaporization enthalpy, Δ_lg_*H*_m_^0^, association enthalpy, Δ_A_*H*_m_^0^ for some ionic liquids; Table S6. The estimated surface tension, *γ*_(est)_, experimental surface tension, *γ*_(exp)_, refractive index, *n*_D_, experimental density *ρ*(exp), molar surface Gibbs energy, *g*_s_, molar refraction, *R*_m_, molar volume, *V* for different ILs and molecular liquids; Table S7. The molar surface entropy, *s*, for some ILs; Density, *ρ*, surface tension, and refractive index, nD measuring methods; citation of ref. [30,39,43,52,54,55,64,65,66,67,68,69,70,71,72,73,74,75,76,77,78,79,80,81,82,83,84,85,86,87,88,89,90,91,92,93,94,95,96,97,98,99,100,101,102,103,104,105,106].

## Sample Availability

Samples of compounds are available from the authors.
