# Supplementary material for: A New Compartmentalized Scale (PN) for Measuring Polarity Applied to Novel Ether-Functionalized Amino Acid Ionic Liquids
Source: Molecules. 2022 May 18;27(10):3231. doi: 10.3390/molecules27103231 (PMC9144623; doi:10.3390/molecules27103231)
Supplement: Supplementary file 1 [file molecules-27-03231-s001.zip › molecules-1717613-supplementary.pdf]

A new compartmentalized scale (P_N) for measuring polarity applied to novel ether-functionalized amino acid ionic liquids

Xu Zheng, Chun Guo, Wenqing Wu, Jing Tong*
College of Chemistry, Liaoning University, Shenyang 110036, P. R. China
*Corresponding author, Email: tongjinglnu@sina.com

Supporting Information

Section A ^{1}H NMR Spectra

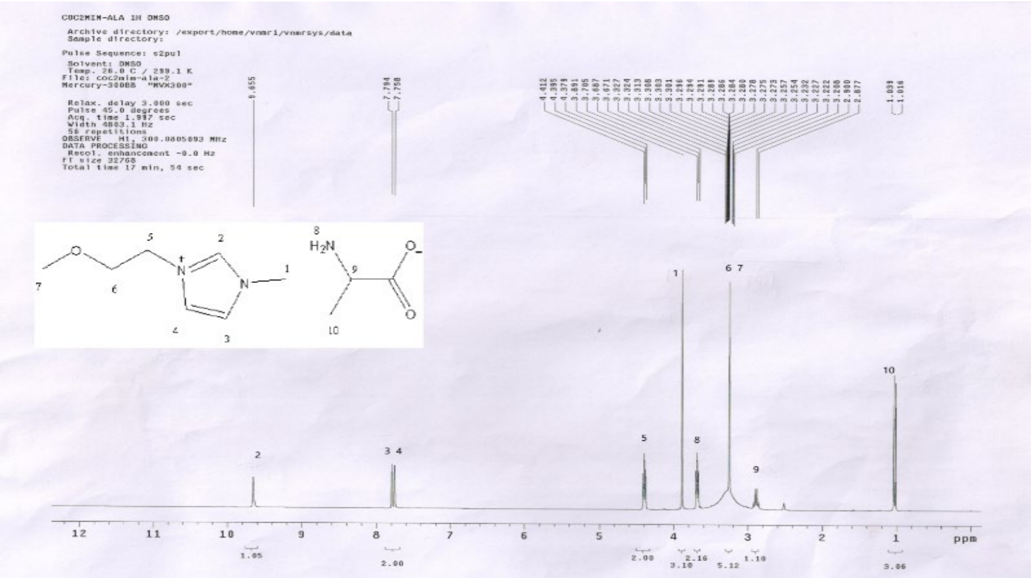

Figure S1. ^{1}H NMR spectrum of IL [COC$_2$mim][Ala].

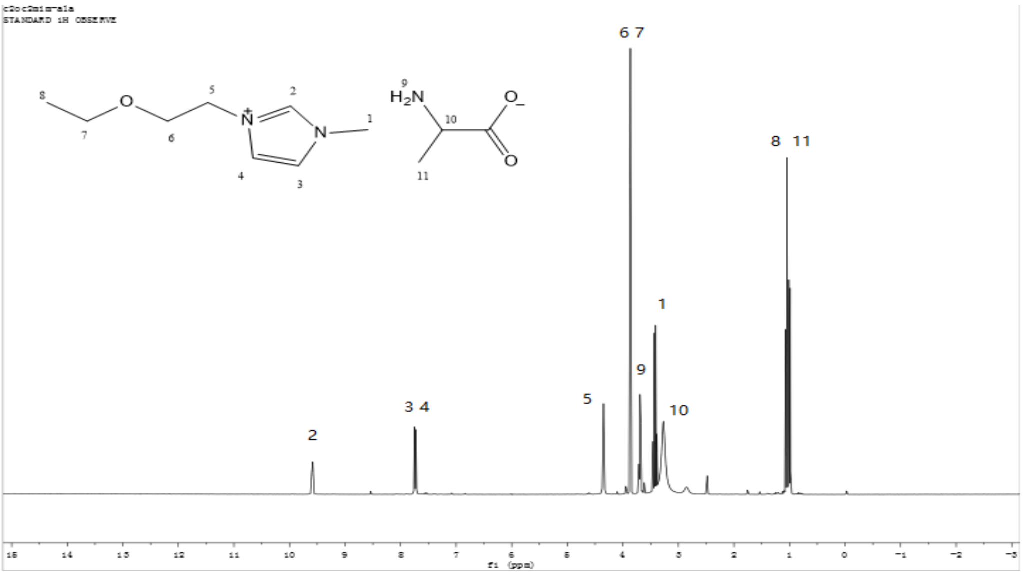

Figure S2. ^{1}H NMR spectrum of IL [C_2OC_2mim][Ala].

Section B ^{13}C NMR Spectra

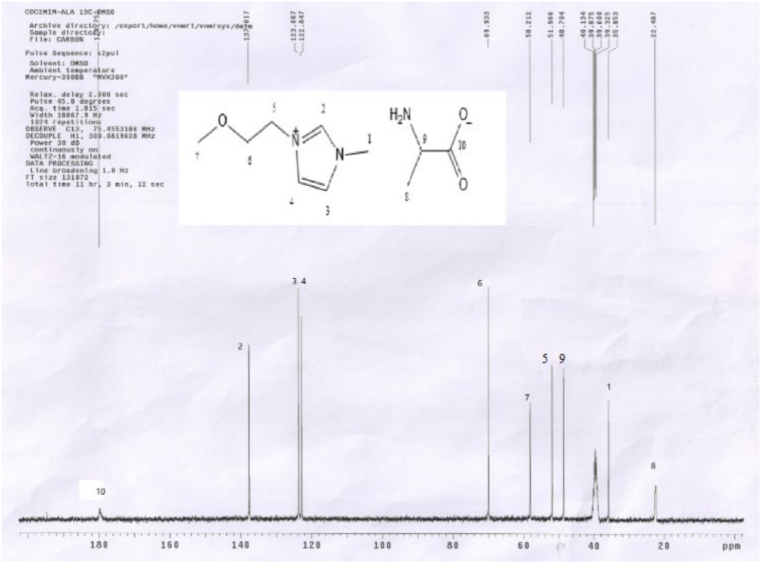

Figure S3. ^{13}C NMR spectrum of IL [COC_2mim][Ala].

.

.

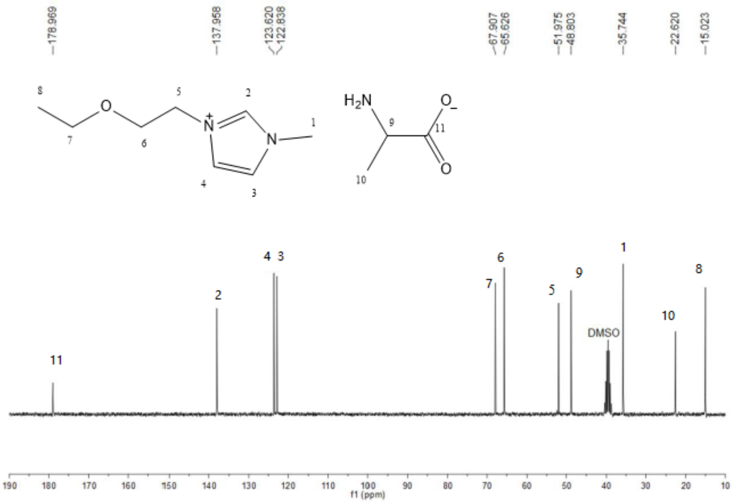

Figure S4. ^{13}C NMR spectrum of IL [C_2OC_2mim][Ala].

Section C

Table S1. Densities of Ionic Liquids Containing Various Amounts of Water at pressure p = 0.1 MPa.

			density ρ / (g·cm^{-3})				
T /K	10^3w_2=4.720	10^3w_2=7.740	10^3w =10.92	10^3w_2=13.76	10^3w_2=16.78	10^3w_2=0	r^2	$sd\times10^5$
				[C_1OC_2mim][Ala]				
288.15	1.16034	1.16012	1.15984	1.15962	1.15937	1.16073	0.9991	1.13
293.15	1.15705	1.15683	1.15656	1.15631	1.15609	1.15744	0.9985	1.50
298.15	1.15385	1.15362	1.15335	1.15315	1.15289	1.15423	0.9990	1.18
303.15	1.15041	1.15018	1.14991	1.14968	1.14939	1.15082	0.9979	1.83
308.15	1.14698	1.14672	1.14648	1.14621	1.14595	1.14739	0.9986	1.53
313.15	1.14353	1.14331	1.14304	1.14278	1.14252	1.14395	0.9980	1.80
318.15	1.14007	1.13984	1.13957	1.13932	1.13905	1.14049	0.9988	1.43
323.15	1.13661	1.13637	1.13610	1.13586	1.13557	1.13703	0.9985	1.61
328.15	1.13324	1.13301	1.13274	1.13252	1.13223	1.13365	0.9980	1.82
T / K	10^3w_2=6.400	10^3w_2=9.600	10^3w_2=12.00	10^3w_2=14.10	10^3w_2=16.90	10^3w_2=0	r^2	$sd\times10^5$
				[C_2OC_2mim][Ala]				
288.15	1.13786	1.13757	1.13732	1.13712	1.13685	1.13849	0.9995	0.892
293.15	1.13415	1.13382	1.13361	1.13341	1.13314	1.13475	0.9992	1.08
298.15	1.13127	1.13098	1.13073	1.13053	1.13026	1.13190	0.9995	0.892
303.15	1.12792	1.12764	1.12739	1.12719	1.12692	1.12854	0.9991	1.16
308.15	1.12451	1.12422	1.12397	1.12377	1.1235	1.12514	0.9995	0.892
313.15	1.12111	1.12082	1.12056	1.12036	1.12008	1.12175	0.9991	1.17
318.15	1.11773	1.11743	1.11717	1.11696	1.11668	1.11838	0.9994	0.984

.

323.15	1.11453	1.11423	1.11396	1.11374	1.11345	1.11521	0.9989	1.37
328.15	1.11099	1.11068	1.11042	1.1102	1.10991	1.11166	0.9995	0.892

w_2: mass fraction of water; r^2: correlation coefficient square; *sd*: standard deviation; Standard uncertainties *u* are $u(T)$=0.02 K, $u(p)$=10 kPa, $u(w_2)$=0.0001; the expanded uncertainty $U(\rho)$ = 0.002 g·cm^{-3} with 0.95 level of confidence($k \approx 2$).

Table S2. Surface Tensions of Ionic Liquids Containing Various Amounts of Water at pressure p = 0.1 MPa

	surface tension γ / (mJ·m^{-2})							
T / K	10^3w_2=4.720	10^3w_2=7.740	10^3w =10.92	10^3w_2=13.76	10^3w_2=16.78	10^3w_2=0	r^2	sd×10^2
				[C_1OC_2mim][Ala]				
288.15	52.2	52.6	53.0	53.3	53.7	51.6	0.998	2.33
293.15	51.8	52.1	52.5	52.9	53.3	51.2	0.995	4.19
298.15	51.5	51.9	52.3	52.6	53.0	50.9	0.998	2.33
303.15	51.1	51.5	51.8	52.2	52.6	50.5	0.995	4.07
308.15	50.7	51.0	51.5	51.8	52.3	50.0	0.991	5.95
313.15	50.3	50.7	51.1	51.4	51.9	49.7	0.999	4.62
318.15	49.9	50.3	50.7	51.0	51.5	49.3	0.994	4.62
323.15	49.5	49.9	50.3	50.7	51.2	48.8	0.996	4.22
328.15	49.1	49.5	49.9	50.3	50.8	48.4	0.996	4.22
T / K	10^3w_2=6.400	10^3w_2=9.600	10^3w_2=12.00	10^3w_2=14.10	10^3w_2=16.90	10^3w_2=0	r^2	sd×10^2
				[C_2OC_2mim][Ala]				
288.15	50.3	50.7	50.9	51.2	51.5	49.6	0.995	3.22
293.15	50.0	50.4	50.7	50.9	51.2	49.3	0.996	2.92
298.15	49.6	50.0	50.3	50.5	50.8	48.9	0.996	2.92
303.15	49.2	49.6	49.8	50.1	50.4	48.5	0.995	3.22
308.15	48.7	49.0	49.3	49.5	49.8	48.0	0.998	2.03
313.15	48.3	48.6	48.9	49.1	49.4	47.6	0.998	2.03
318.15	47.9	48.3	48.5	48.8	49.1	47.2	0.995	3.22
323.15	47.4	47.8	48.1	48.3	48.6	46.7	0.996	2.92
328.15	47.0	47.4	47.6	47.9	48.2	46.3	0.995	3.22

w_2: mass fraction of water; r^2: correlation coefficient square; *sd*: standard deviation; Standard uncertainties *u* are $u(T)$=0.02 K, $u(p)$=10 kPa, $u(w_2)$=0.0001; the expanded uncertainty $U(\gamma)$ = 0.3 mJ·m^{-2} with 0.95 level of confidence($k \approx 2$).

Table S3. Refractive Indexes of Ionic Liquids Containing Various Amount of Water at pressure p = 0.1 MPa.

	refractive index n_D							
T / K	10^3w_2=4.720	10^3w_2=7.740	10^3w =10.92	10^3w_2=13.76	10^3w_2=16.78	10^3w_2=0	r^2	sd×10^5
				[C_1OC_2mim][Ala]				
288.15	1.5103	1.5098	1.5092	1.5087	1.5081	1.5112	0.999	2.90
293.15	1.5089	1.5083	1.5077	1.5072	1.5067	1.5097	0.998	3.67
298.15	1.5072	1.5066	1.5061	1.5056	1.5050	1.5080	0.998	3.92
303.15	1.5058	1.5053	1.5047	1.5043	1.5038	1.5066	0.998	3.71
308.15	1.5042	1.5038	1.5034	1.5031	1.5027	1.5048	0.998	2.33

313.15	1.5031	1.5026	1.5022	1.5018	1.5013	1.5038	0.997	3.82
318.15	1.5014	1.5010	1.5006	1.5003	1.5000	1.5019	0.995	4.06
323.15	1.4998	1.4994	1.4990	1.4987	1.4983	1.5004	0.998	2.33
328.15	1.4985	1.4981	1.4977	1.4973	1.4969	1.4991	1.00	1.17

T/K	10^3w_2=6.400	10^3w_2=9.600	10^3w_2=12.00	10^3w_2=14.10	10^3w_2=16.90	10^3w_2=0	r^2	sd×10^5
				[C2OC2mim][Ala]				
288.15	1.4928	1.4922	1.4916	1.4912	1.4906	1.4942	0.998	4.06
293.15	1.4913	1.4907	1.4902	1.4897	1.4891	1.4927	0.998	4.24
298.15	1.4900	1.4894	1.4888	1.4884	1.4878	1.4914	0.998	4.06
303.15	1.4887	1.4880	1.4875	1.4871	1.4866	1.4899	0.997	4.32
308.15	1.4874	1.4869	1.4864	1.4861	1.4856	1.4885	0.997	3.71
313.15	1.4858	1.4853	1.4849	1.4845	1.4841	1.4869	0.998	2.98
318.15	1.4844	1.4839	1.4836	1.4833	1.4828	1.4854	0.995	4.13
323.15	1.4830	1.4825	1.4822	1.4819	1.4815	1.4839	0.999	2.11
328.15	1.4814	1.4810	1.4806	1.4803	1.4799	1.4824	0.998	2.81

w_2：mass fraction of water; r^2：correlation coefficient square; sd：standard deviation; Standard uncertainties u are $u(T)$=0.02 K, $u(p)$=10 kPa, $u(w_2)$=0.0001; the expanded uncertainty $U(n_D)$ =0.003 with 0.95 level of confidence($k \approx$ 2).

Table S4. The values of molar volume, V/cm^3·mol^{-1} and molecular volume, V_m/nm^3 for the ILs [C$_n$OC$_2$mim][Ala](n=1, 2)

T/K	288.15	293.15	298.15	303.15	308.15	313.15	318.15	323.15	328.15
				[C1OC2mim][Ala]					
V/cm^3·mol^{-1}	197.5	198.1	198.6	199.2	199.8	200.4	201.0	201.6	202.2
V_m/nm^3	0.3281	0.3291	0.3300	0.3309	0.3319	0.3329	0.3339	0.3350	0.3360
				[C2OC2mim][Ala]					
V/cm^3·mol^{-1}	213.7	214.4	215.0	215.6	216.2	216.9	217.5	218.2	218.9
V_m/nm^3	0.3550	0.3562	0.3571	0.3581	0.3592	0.3603	0.3614	0.3624	0.3636

Table S5. Molecular volume, V_m, standard molar entropy, S^0, lattice energy, U_{POT}, vaporization enthalpy, $\Delta_l^g H_m^0$, association enthalpy, $\Delta_A H_m^0$ for some ionic liquids.

ILs	V_m/nm^3	$S^0_{(298)}$/J·K^{-1}·mol^{-1}	U_{POT}/ kJ·mol^{-1}	$\Delta_l^g H_m^0$/kJ·mol^{-1}	$\Delta_A H_m^0$/kJ·mol^{-1}
[C1OC2mim][Ala]	0.3300	441	443	164.1	-278.9
[C2OC2mim][Ala]	0.3571	475	435	165.1	-269.9
[C4mim]OAc	0.3143[1]	421	449	134.8[2]	-314
[C6mim]OAc	0.3695[1]	490	431	140.7[2]	-290
[C2mim]BF4	0.2569[3]	350	473	132.7[4]	-340
[C4mim]BF4	0.3124[3]	419	450	137.8[4]	-312
[C3mim]Pro	0.3128[5]	419	449	123.9[5]	-326

.

$[C_4mim]Pro$	0.3405[5]	454	440	126.8[5]	-313
$[C_5mim]Pro$	0.3683[5]	489	431	130.3[5]	-301
$[C_6mim]Pro$	0.3962[5]	523	423	136.5[5]	-287
$[C_2mim]Lact$	0.2804[6]	379	462	138.8[7]	-323
$[C_5mim]Lact$	0.3639[6]	483	432	145.4[7]	-287
$[C_2mmim]NTf_2$	0.4516[8]	592	410	156.5[9]	-253
$[C_4mmim]NTf_2$	0.5075[8]	662	398	160.5[9]	-237
$[C_2mim]NTf_2$	0.4332[10]	569	414	132.7[11]	-281
$[C_4mim]NTf_2$	0.4904[10]	641	401	137.8[11]	-264
$[C_6mim]NTf_2$	0.5586[10]	726	389	142.3[11]	-246
$[C_8mim]NTf_2$	0.6027[10]	781	382	147.0[11]	-235
$[C_4mim]PF_6$	0.3451[11]	460	438	154.8[12]	-283
$[C_6mim]PF_6$	0.4010[11]	529	422	139.8[11]	-282
$[C_8mim]PF_6$	0.4613[11]	605	407	144.3[11]	-263
$[C_{10}mim]PF_6$	0.5131[11]	669	397	135.9[11]	-261

Table S6. The estimated surface tension, $\gamma_{(est)}$, experimental surface tension, $\gamma_{(exp)}$, refractive index, n_D, experimental density $\rho_{(exp)}$, molar surface Gibbs energy, g_s, molar refraction, R_m, molar volume, V for different ILs and molecular liquids.

T/K	$\gamma_{(est)}$ /mJ·m^{-2}	$\gamma_{(exp)}$ /mJ·m^{-2}	$n_{D(exp)}$	$\rho_{(exp)}$/g·cm^{-3}	g_s /J·mol^{-1}	R_m cm^3·mol^{-1}	V /cm^3·mol^{-1}	$g_{s(est)}$ J/mol
			Ionic liquids					
			$[C_2mim]OAC$[1]					
298.15	38.2	38.1	1.4938	1.10190	9263	44.95	154.5	9280
303.15	37.8	37.8	1.4926	1.09882	9207	44.99	154.9	9205
308.15	37.4	37.5	1.4914	1.09578	9151	45.02	155.3	9130
313.15	37.0	37.1	1.4903	1.09273	9070	45.06	155.8	9055
318.15	36.7	36.6	1.4890	1.08968	8964	45.08	156.2	8980
323.15	36.3	36.3	1.4876	1.08665	8907	45.10	156.6	8904
328.15	35.9	35.9	1.4863	1.08363	8826	45.12	157.1	8829

333.15	35.5	35.5	1.4854	1.08064	8743	45.17	157.5	8754
338.15	35.2	35.2	1.4844	1.07767	8685	45.22	157.9	8679
			[C_3mim]OAC[1]					
298.15	36.7	36.8	1.4902	1.07942	9562	49.36	170.7	9540
303.15	36.3	36.3	1.4890	1.07627	9451	49.41	171.2	9452
308.15	35.9	35.8	1.4880	1.07315	9338	49.46	171.7	9364
313.15	35.5	35.4	1.4868	1.07003	9252	49.50	172.2	9275
318.15	35.1	35.1	1.4858	1.06693	9191	49.56	172.7	9187
323.15	34.7	34.7	1.4845	1.06386	9104	49.59	173.2	9099
328.15	34.3	34.4	1.4835	1.06077	9043	49.65	173.7	9011
333.15	33.9	33.9	1.4824	1.05773	8929	49.69	174.2	8922
338.15	33.5	33.4	1.4811	1.05466	8814	49.72	174.7	8834
			[C_4mim]OAC[1]					
298.15	35.3	35.2	1.4869	1.04740	9800	54.43	189.3	9831
303.15	34.9	34.9	1.4857	1.04437	9735	54.48	189.8	9739
308.15	34.5	34.6	1.4845	1.04137	9670	54.52	190.4	9648
313.15	34.1	34.2	1.4831	1.03827	9577	54.55	191.0	9557
318.15	33.8	33.9	1.4820	1.03521	9512	54.60	191.5	9466
323.15	33.4	33.3	1.4807	1.03219	9362	54.63	192.1	9375
328.15	32.9	32.8	1.4795	1.02918	9239	54.68	192.6	9284
333.15	32.6	32.6	1.4782	1.02625	9200	54.71	193.2	9193
338.15	32.2	32.2	1.4771	1.02328	9105	54.76	193.8	9102
			[C_5mim]OAC[1]					
298.15	34.2	34.1	1.4845	1.03013	10047	59.01	206.1	10076
303.15	33.8	33.8	1.4834	1.02705	9978	59.07	206.7	9975
308.15	33.4	33.4	1.4822	1.02400	9880	59.12	207.3	9875
313.15	33.0	33.1	1.4809	1.02098	9810	59.16	207.9	9774
318.15	32.6	32.6	1.4797	1.01794	9681	59.21	208.5	9673
323.15	32.2	32.2	1.4785	1.01491	9582	59.26	209.2	9573

328.15	31.8	31.7	1.4776	1.01185	9452	59.35	209.8	9472
333.15	31.4	31.3	1.4764	1.00888	9351	59.39	210.4	9372
338.15	31.0	31.0	1.4751	1.00588	9280	59.43	211.0	9271
			[C6mim]OAC[1]					
298.15	33.1	33.0	1.4829	1.01700	10233	63.54	222.5	10259
303.15	32.7	32.7	1.4816	1.01393	10161	63.59	223.2	10150
308.15	32.2	32.3	1.4802	1.01088	10057	63.62	223.9	10041
313.15	31.8	31.9	1.4789	1.00785	9952	63.67	224.6	9933
318.15	31.4	31.4	1.4776	1.00490	9815	63.70	225.2	9824
323.15	31.0	31.0	1.4763	1.00188	9710	63.75	225.9	9716
328.15	30.6	30.6	1.4750	0.99888	9604	63.79	226.6	9607
333.15	30.2	30.2	1.4735	0.99589	9497	63.81	227.2	9499
338.15	29.8	29.8	1.4722	0.99288	9390	63.85	227.9	9390
			[C2mim]Lact[12]					
288.15	49.5	49.5	1.5026	1.1929	12720	49.59	167.86	12723
293.15	49.1	49.2	1.5014	1.1898	12665	49.62	168.30	12653
298.15	48.8	48.8	1.5001	1.1861	12588	49.66	168.82	12583
303.15	48.4	48.4	1.4987	1.1834	12504	49.66	169.21	12512
308.15	48.1	48.0	1.4977	1.1801	12423	49.71	169.68	12442
313.15	47.7	47.7	1.4961	1.1764	12372	49.73	170.21	12371
318.15	47.3	47.4	1.4950	1.1734	12315	49.77	170.65	12301
323.15	47.0	47.0	1.4936	1.1704	12232	49.77	171.09	12231
328.15	46.6	46.6	1.4926	1.1671	12151	49.83	171.57	12160
333.15	46.3	46.3	1.4913	1.1636	12096	49.87	172.09	12090
			[C5mim]Lact[12]					
288.15	43.1	43.1	1.4940	1.1126	13175	63.41	217.80	13174
293.15	42.7	42.7	1.4928	1.1090	13081	63.48	218.50	13085
298.15	42.4	42.4	1.4915	1.1060	13013	63.51	219.10	12996
303.15	42.0	42.0	1.4901	1.1027	12916	63.54	219.75	12907

308.15	41.6	41.5	1.4891	1.0994	12787	63.62	220.41	12819
313.15	41.2	41.2	1.4875	1.0953	12727	63.69	221.24	12730
318.15	40.9	40.9	1.4864	1.0929	12652	63.70	221.72	12641
323.15	40.5	40.5	1.4850	1.0899	12552	63.72	222.33	12552
328.15	40.1	40.1	1.4841	1.0864	12454	63.82	223.05	12463
333.15	39.8	39.8	1.4827	1.0833	12385	63.85	223.69	12374
			$[C_2mmim]NTf_2$[8]					
298.15	33.5	33.5	1.4280	1.49107	11872	69.95	271.9	11863
303.15	33.3	33.3	1.4265	1.48624	11827	69.96	272.8	11812
308.15	33.0	33.0	1.4250	1.48142	11746	69.97	273.6	11762
313.15	32.8	32.8	1.4235	1.47662	11700	69.98	274.5	11711
318.15	32.6	32.6	1.4220	1.47183	11654	69.99	275.4	11661
323.15	32.4	32.4	1.4205	1.46706	11608	70.00	276.3	11611
328.15	32.2	32.2	1.4190	1.46231	11561	70.01	277.2	11560
333.15	32.0	32.0	1.4175	1.45757	11514	70.02	278.1	11510
338.15	31.8	31.8	1.4161	1.45284	11467	70.04	279.0	11459
			$[C_4mmim]NTf_2$[8]					
298.15	32.4	32.5	1.4325	1.41852	12450	79.32	305.5	12433
303.15	32.2	32.2	1.4311	1.41391	12362	79.36	306.5	12377
308.15	32.0	32.0	1.4298	1.40929	12312	79.41	307.5	12322
313.15	31.8	31.8	1.4286	1.40468	12261	79.48	308.5	12266
318.15	31.6	31.6	1.4273	1.40007	12211	79.53	309.5	12210
323.15	31.4	31.4	1.4260	1.39548	12160	79.57	310.6	12154
328.15	31.2	31.2	1.4247	1.39090	12109	79.62	311.6	12099
333.15	31.0	31.0	1.4232	1.38633	12058	79.64	312.6	12043
338.15	30.8	30.7	1.4218	1.37178	11968	79.67	313.6	11987
			$[C_2mim]BF_4$[3]					
298.15	50.1	50.1	1.4121	1.2798	12192	38.50	154.7	12180
303.15	49.7	49.7	1.4109	1.2777	12108	38.46	154.9	12112

308.15	49.4	49.3	1.4099	1.2752	12026	38.46	155.3	12043
313.15	49.0	49.0	1.4090	1.2724	11970	38.47	155.6	11975
318.15	48.7	48.7	1.4080	1.2700	11912	38.46	155.9	11907
323.15	48.3	48.4	1.4069	1.2675	11854	38.44	156.2	11839
328.15	48.0	48.0	1.4057	1.2649	11772	38.42	156.5	11770
333.15	47.6	47.6	1.4047	1.2620	11692	38.42	156.9	11702
338.15	47.3	47.3	1.4036	1.2593	11635	38.41	157.2	11634
[C_3mim]BF_4[3]								
298.15	47.1	47.0	1.4165	1.2361	12252	43.09	171.5	12288
308.15	46.4	46.4	1.4142	1.2312	12128	43.05	172.2	12128
313.15	46.0	46.1	1.4132	1.2288	12065	43.04	172.5	12048
318.15	45.7	45.8	1.4120	1.2268	11999	43.00	172.8	11968
323.15	45.3	45.4	1.4110	1.2241	11912	43.00	173.2	11888
328.15	45.0	45.0	1.4098	1.2218	11822	42.97	173.5	11808
333.15	44.6	44.5	1.4086	1.2193	11707	42.95	173.9	11728
338.15	44.2	44.1	1.4075	1.2167	11618	42.94	174.2	11647
[C_4mim]BF_4[3]								
298.15	44.7	44.7	1.4211	1.2015	12393	47.72	188.1	12398
303.15	44.3	44.3	1.4200	1.1991	12299	47.70	188.5	12309
308.15	44.0	44.0	1.4192	1.1977	12225	47.68	188.7	12220
313.15	43.6	43.6	1.4182	1.1955	12129	47.67	189.1	12131
318.15	43.2	43.3	1.4172	1.1932	12061	47.66	189.4	12043
323.15	42.9	42.9	1.4162	1.1912	11963	47.64	189.7	11954
328.15	42.5	42.5	1.4151	1.1885	11869	47.63	190.2	11865
333.15	42.1	42.1	1.4140	1.1865	11770	47.60	190.5	11776
338.15	41.8	41.7	1.4130	1.1840	11675	47.60	190.9	11687
[C_5mim]BF_4[3]								
298.15	42.9	42.9	1.4238	1.1719	12589	52.25	204.8	12579
303.15	42.5	42.5	1.4228	1.1696	12488	52.24	205.2	12481

308.15	42.1	42.1	1.4216	1.1675	12385	52.21	205.6	12383
313.15	41.7	41.7	1.4206	1.1657	12280	52.18	205.9	12285
318.15	41.3	41.3	1.4197	1.1633	12179	52.19	206.4	12187
323.15	41.0	40.9	1.4185	1.1613	12075	52.15	206.7	12089
328.15	40.6	40.5	1.4175	1.1592	11971	52.13	207.1	11991
333.15	40.2	40.2	1.4164	1.1572	11896	52.10	207.4	11893
338.15	39.8	39.9	1.4153	1.1551	11822	52.08	207.8	11795
			$[C_6mim]BF_4$[3]					
298.15	41.0	41.0	1.4270	1.1463	12681	56.91	221.7	12693
303.15	40.6	40.7	1.4260	1.1441	12604	56.90	222.1	12586
308.15	40.2	40.3	1.4249	1.1420	12496	56.88	222.5	12478
313.15	39.9	39.9	1.4237	1.1403	12384	56.82	222.8	12371
318.15	39.5	39.4	1.4226	1.1383	12243	56.79	223.2	12263
323.15	39.1	39.0	1.4215	1.1361	12134	56.77	223.6	12156
328.15	38.7	38.6	1.4204	1.1345	12021	56.72	224.0	12049
333.15	38.3	38.3	1.4191	1.1322	11944	56.68	224.4	11941
338.15	37.9	38.0	1.4179	1.1302	11864	56.64	224.8	11834
			$[C_3mim]Gly$[13]					
293.15	45.9	45.9	1.5091	1.1391	12123	52.24	174.9	12131
298.15	45.6	45.6	1.5069	1.1358	12067	52.20	175.4	12068
303.15	45.3	45.3	1.5050	1.1331	12007	52.16	175.8	12006
308.15	45.0	45.0	1.5031	1.1299	11950	52.14	176.3	11943
313.15	44.7	44.7	1.5005	1.1272	11889	52.03	176.8	11881
318.15	44.4	44.4	1.4990	1.1246	11828	52.02	177.2	11818
323.15	44.1	44.0	1.4973	1.1220	11739	51.99	177.6	11756
			$[C_4mim]Gly$[13]					
293.15	43.9	43.8	1.5091	1.1142	12285	57.16	191.4	12299
298.15	43.5	43.5	1.5085	1.1109	12225	57.28	192.0	12229
303.15	43.2	43.2	1.5068	1.1078	12163	57.28	192.5	12159

308.15	42.9	42.9	1.5049	1.1047	12101	57.25	193.1	12088
313.15	42.5	42.6	1.5038	1.1016	12039	57.31	193.6	12018
318.15	42.2	42.2	1.5029	1.0986	11948	57.38	194.1	11948
323.15	41.9	41.8	1.5019	1.0955	11857	57.44	194.7	11878
			[C5mim]Gly[14]					
293.15	41.8	41.8	1.5080	1.0979	12353	61.72	207.0	12361
298.15	41.5	41.5	1.5066	1.0947	12289	61.75	207.6	12290
303.15	41.2	41.2	1.5053	1.0918	12221	61.78	208.2	12219
308.15	40.9	40.9	1.5040	1.0889	12154	61.81	208.7	12148
313.15	40.6	40.6	1.5028	1.0863	12084	61.83	209.2	12077
318.15	40.3	40.3	1.5013	1.0836	12015	61.83	209.8	12006
323.15	40.0	39.9	1.5001	1.0802	11920	61.90	210.4	11935
			[C6mim]Gly[14]					
293.15	41.3	41.3	1.4959	1.0788	12852	65.34	223.7	12838
298.15	40.9	40.9	1.4944	1.0755	12754	65.37	224.4	12754
303.15	40.6	40.5	1.4930	1.0725	12653	65.39	225.0	12671
308.15	40.2	40.2	1.4919	1.0701	12578	65.42	225.5	12587
313.15	39.9	39.9	1.4905	1.0671	12507	65.44	226.2	12504
318.15	39.6	39.6	1.4893	1.0646	12433	65.46	226.7	12421
323.15	39.2	39.2	1.4876	1.0608	12336	65.50	227.5	12337
			[C3mim]Pro[15]					
288.15	37.7	37.7	1.4906	1.05883	10420	54.19	187.2	10428
293.15	37.5	37.5	1.4895	1.05562	10386	54.26	187.8	10389
298.15	37.3	37.3	1.4884	1.05260	10350	54.31	188.4	10350
303.15	37.1	37.1	1.4874	1.04960	10314	54.37	188.9	10311
308.15	36.9	36.9	1.4861	1.04644	10279	54.41	189.5	10272
313.15	36.7	36.7	1.4851	1.04337	10243	54.47	190.0	10233
318.15	36.4	36.5	1.4840	1.04034	10207	54.52	190.6	10194
323.15	36.3	36.2	1.4829	1.03724	10144	54.58	191.1	10156

328.15	36.0	36.0	1.4818	1.03428	10107	54.63	191.7	10117
		[C4mim]Pro[15]						
288.15	36.5	36.5	1.4880	1.04125	10677	58.74	203.9	10685
293.15	36.3	36.3	1.4870	1.03819	10639	58.81	204.5	10643
298.15	36.1	36.1	1.4861	1.03527	10601	58.88	205.1	10600
303.15	35.9	35.9	1.4851	1.03225	10563	58.95	205.7	10557
308.15	35.7	35.7	1.4839	1.02931	10524	59.00	206.2	10514
313.15	35.4	35.5	1.4830	1.02628	10485	59.07	206.9	10471
318.15	35.2	35.2	1.4820	1.02321	10418	59.15	207.5	10428
323.15	35.0	35.0	1.4809	1.02014	10379	59.21	208.1	10385
328.15	34.8	34.8	1.4797	1.01706	10341	59.26	208.7	10343
		[C5mim]Pro[15]						
288.15	35.4	35.4	1.4862	1.02695	10907	63.30	220.4	10917
293.15	35.2	35.2	1.4854	1.02389	10867	63.39	221.0	10871
298.15	35.0	35.0	1.4843	1.02051	10829	63.48	221.8	10826
303.15	34.8	34.8	1.4830	1.01768	10787	63.52	222.4	10780
308.15	34.5	34.6	1.4830	1.01471	10746	63.70	223.0	10735
313.15	34.4	34.4	1.4810	1.01175	10704	63.66	223.7	10689
318.15	34.1	34.1	1.4798	1.00872	10632	63.71	224.4	10644
323.15	33.9	33.9	1.4787	1.00572	10591	63.78	225.0	10598
328.15	33.7	33.7	1.4774	1.00269	10550	63.83	225.7	10553
		[C6mim]Pro[15]						
288.15	34.3	34.3	1.4842	1.01343	11098	67.87	237.2	11106
293.15	34.1	34.1	1.4834	1.01047	11054	67.97	237.9	11059
298.15	33.9	33.9	1.4823	1.00735	11012	68.05	238.6	11012
303.15	33.7	33.7	1.4810	1.00439	10969	68.10	239.3	10965
308.15	33.5	33.5	1.4800	1.00141	10925	68.18	240.0	10918
313.15	33.3	33.3	1.4790	0.99843	10882	68.26	240.7	10870
318.15	33.0	33.1	1.4776	0.99539	10838	68.31	241.5	10823

323.15	32.9	32.8	1.4767	0.99240	10762	68.39	242.2	10776
328.15	32.6	32.6	1.4755	0.98939	10718	68.45	242.9	10729
			[C3mim]Ser[16]					
298.15	46.8	46.8	1.5179	1.18151	13235	58.72	193.8	13245
303.15	46.6	46.6	1.5165	1.17834	13201	58.73	194.3	13199
308.15	46.3	46.4	1.5153	1.17519	13172	58.80	194.9	13153
313.15	46.1	46.1	1.5141	1.17203	13109	58.84	195.4	13107
318.15	45.9	45.8	1.5131	1.16888	13046	58.89	195.9	13062
323.15	45.6	45.6	1.5123	1.16567	13015	58.99	196.5	13016
328.15	45.4	45.4	1.5112	1.16260	12980	59.04	197.0	12970
333.15	45.1	45.1	1.5100	1.15949	12916	59.07	197.5	12924
338.15	44.9	44.9	1.5086	1.15642	12881	59.08	198.0	12878
			[C4mim]Ser[16]					
298.15	45.4	45.4	1.5143	1.15604	13554	63.31	210.2	13563
303.15	45.2	45.2	1.5135	1.15282	13520	63.41	210.8	13516
308.15	44.9	45.0	1.5126	1.14961	13485	63.50	211.4	13469
313.15	44.7	44.7	1.5114	1.14645	13421	63.55	212.0	13423
318.15	44.5	44.4	1.5106	1.14331	13352	63.62	212.5	13376
323.15	44.2	44.3	1.5096	1.14008	13347	63.69	213.1	13329
328.15	44.0	44.0	1.5091	1.13691	13281	63.82	213.7	13283
333.15	43.8	43.8	1.5077	1.13381	13246	63.85	214.3	13236
338.15	43.5	43.5	1.5069	1.13064	13179	63.94	214.9	13189
			Molecular liquids					
			Chloroform					
283.15	28.6	28.6	1.45189[17]	1.5073[17]	4454	21.36	79.2	4457
293.15	27.3	27.3	1.44589[17]	1.4884[17]	4287	21.38	80.2	4288
298.15	26.6	26.7	1.44207[18]	1.4795[19]	4211	21.35	80.7	4203
303.15	26.0	26.0	1.43987[17]	1.4694 [17]	4117	21.41	81.2	4118
313.15	24.7	24.7	1.43386 [17]	1.45 [17]	3946	21.43	82.3	3949

		Pyridine						
293	37.2	37.2	1.51016[20]	0.9832[21]	5857	24.07	80.5	5860
298	36.5	36.6	1.5083[22]	0.97824[23]	5781	24.12	80.9	5774
308	35.2	35.2	1.5044 [22]	0.96840[23]	5597	24.20	81.7	5603
313	34.6	34.6	1.4985[24]	0.9637[25]	5519	24.08	82.1	5517
		1-hexanol						
293.15	26.2	26.2	1.4172 [26]	0.81875[27]	5525	31.40	124.8	5526
298.15	25.8	25.8	1.4157 [26]	0.81523[27]	5455	31.43	125.3	5457
303.15	24.4	25.4	1.4137 [26]	0.81160[27]	5388	31.44	125.9	5387
308.15	25.0	25.0	1.4144 [26]	0.80800[27]	5320	31.63	126.5	5318
313.15	24.6	24.6	1.4098 [26]	0.80460[28]	5249	31.45	127.0	5248
323.15	23.8	23.8	1.4057 [26]	0.79750[28]	5107	31.45	128.1	5109
		benzyl alcohol						
298.15	34.8	34.8	1.5831[29]	1.04353[30]	6482	34.64	103.6	6485
303.15	34.0	34.1	1.5363[31]	1.037 [30]	6381	32.53	104.3	6375
308.15	33.4	33.4	1.5342 [32]	1.03366 [30]	6262	32.53	104.6	6264
		Cyclohexanol						
298.15	32.9	32.9	1.4642[33]	0.94496[34]	6223	29.26	106.0	6223
303.15	32.4	32.4	1.4627[35]	0.9412[35]	6144	29.29	106.4	6143
308.15	31.9	31.9	1.4608[35]	0.9377[35]	6064	29.30	106.8	6064
		isopropyl alcohol						
293.15	21.3	21.3	1.37709[36]	0.7851[37]	3241	17.61	76.5	3239
303.15	20.5	20.5	1.37487[38]	0.77663[37]	3144	17.71	77.4	3145
308.15	20.1	20.1	1.3721[39]	0.77187[40]	3096	17.70	77.9	3098
323.15	19.0	19.0	1.3744[41]	0.75868[37]	2959	18.11	79.2	2957
		1-propanol						
293.15	23.7	23.7	1.3851[26]	0.803769[42]	3553	17.52	74.8	3549
298.15	23.3	23.3	1.3832[26]	0.799762[42]	3502	17.54	75.1	3503
303.15	22.9	22.9	1.3814[26]	0.795722[42]	3454	17.55	75.5	3458

.

308.15	22.5	22.5	1.3793[26]	0.791639[42]	3406	17.56	75.9	3412
313.15	22.2	22.2	1.3774[26]	0.78765[43]	3372	17.57	76.3	3366
		Ethanol						
293.15	22.4	22.4	1.3615[26]	0.7897 [44]	2591	11.24	50.7	2594
298.15	22.0	22.0	1.3593[26]	0.7858[45]	2555	11.23	51.0	2554
303.15	21.5	21.6	1.3573[26]	0.78109[44]	2518	11.25	51.3	2514
313.15	20.7	20.7	1.3533[26]	0.77234[44]	2432	11.26	51.9	2435
318.15	20.4	20.3	1.3512[26]	0.768042[46]	2394	11.26	52.2	2395
323.15	19.9	19.9	1.3491[26]	0.762564[46]	2356	11.28	52.5	2355
		Methanol						
293.15	22.5	22.5	1.3284[26]	0.79128[44]	2241	8.22	40.5	2241
298.15	22.1	22.1	1.3267[26]	0.78701[47]	2208	8.23	40.7	2209
303.15	21.7	21.7	1.3247[26]	0.78184[44]	2179	8.24	41.0	2178
308.15	21.3	21.3	1.3229[26]	0.77710[48]	2146	8.25	41.2	2146
313.15	20.9	20.9	1.3207[26]	0.77230[44]	2116	8.25	41.5	2115
318.15	20.5	20.5	1.3187[26]	0.767326[49]	2085	8.25	41.8	2084
323.15	20.1	20.1	1.3165[26]	0.76257[49]	2051	8.25	42.0	2052

Table S7 The molar surface entropy, *s*, for some ILs

ILs	s/J·mol^{-1}·K^{-1}
[C_2mim][OAc]	15.04
[C_3mim][OAc]	17.66
[C_4mim][OAc]	18.20
[C_5mim][OAc]	20.12
[C_6mim][OAc]	21.71
[C_2mmim]NTf_2	10.08
[C_4mmim]NTf_2	11.14
[C_2mim]BF_4	13.66
[C_3mim]BF_4	16.02
[C_4mim]BF_4	17.78
[C_5mim]BF_4	19.59
[C_6mim]BF_4	21.48
[C_3mim]Gly	12.49
[C_4mim]Gly	14.01
[C_5mim]Gly	14.17

.

[C_6mim]Gly	16.68
[C_3mim]Pro	7.78
[C_4mim]Pro	8.57
[C_5mim]Pro	9.10
[C_6mim]Pro	9.44

Density, ρ, surface tension, γ and refractive index, n_D measuring methods

[C_nOC_2mim][Ala](n=1, 2) tend to form hydrogen bonds with water. To remove the impact of water, standard addition method (SAM) was selected to measuring ρ, γ and n_D [50]. Taking the initial water content into account, a series samples of [C_nOC_2mim][Ala](n=1, 2) with different water contents were prepared freshly. Each sample was processed on an electronic balance (A104) with calibration of air buoyancy

An Anton Paar DMA 4500 oscillating U-tube densitometer was picked to determine the density of the samples. Firstly, the density of pure water were measured and the results were in good agreement with the literature[51], within the experimental error of ±0.00002 g·cm^{-3}. The temperature in the cell was controlled by a solid-state thermostat to±0.01 K. Then the apparatus was used for [C_nOC_2mim][Ala](n=1, 2).

The tensiometer of the forced bubble method (DP-AW type produced by Sang Li Electronic Co.) was used to measure the surface tension. Firstly, the surface tension of pure water were determined, and the results were in good agreement with the literature[51], within the experimental error ±0.1 mJ·m^{-2}. Then the apparatus was used for [C_nOC_2mim][Ala](n=1, 2).

An Abbe refractometer was employed to measure the refractive indexes. Firstly, the refractive indexes of pure water were measured, and the results were in good agreement with the literature, within the experimental error ±0.0001[51]. Then the apparatus was used for [C_nOC_2mim][Ala](n=1, 2).

References

1. Ma, X.-X.; Wei, J.; Zhang, Q.-B.; Tian, F.; Feng, Y.-Y.; Guan, W., Prediction of Thermophysical Properties of Acetate-Based Ionic Liquids Using Semiempirical Methods. *Industrial & Engineering Chemistry Research* **2013,** 52, (27), 9490-9496.
2. Wei, J.; Bu, X.; Guan, W.; Xing, N.; Fang, D.; Wu, Y., Measurement of vaporization enthalpy by isothermogravimetrical method and prediction of the polarity for 1-alkyl-3-methylimidazolium acetate { C(n)mim OAc (n=4, 6)} ionic liquids. *Rsc Advances* **2015,** 5, (86), 70333-70338.
3. Xu, W.-G.; Li, L.; Ma, X.-X.; Wei, J.; Duan, W.-B.; Guan, W.; Yang, J.-Z., Density, surface tension, and refractive index of ionic liquids homologue of 1-alkyl-3-methylimidazolium

tetrafluoroborate [C n mim][BF4](n= 2, 3, 4, 5, 6). *Journal of Chemical & Engineering Data* **2012,** 57, (8), 2177-2184.

4. Verevkin, S. P.; Zaitsau, D. H.; Emel'yanenko, V. N.; Yermalayeu, A. V.; Schick, C.; Liu, H.; Maginn, E. J.; Bulut, S.; Krossing, I.; Kalb, R., Making sense of enthalpy of vaporization trends for ionic liquids: new experimental and simulation data show a simple linear relationship and help reconcile previous data. *The Journal of Physical Chemistry B* **2013,** 117, (21), 6473-6486.
5. Tong, J.; Yang, H. X.; Liu, R. J.; Li, C.; Xia, L. X.; Yang, J. Z., Determination of the Enthalpy of Vaporization and Prediction of Surface Tension for Ionic Liquid 1-Alkyl-3-methylimidazolium Propionate C(n)mim Pro (n=4, 5, 6). *Journal of Physical Chemistry B* **2014,** 118, (45), 12972-12978.
6. Tong, J.; Hong, M.; Liu, C.; Sun, A.; Guan, W.; Yang, J.-Z., Estimation of Properties of Ionic Liquids 1-Alkyl-3-methylimidazolium Lactate Using a Semiempirical Method. *Industrial & Engineering Chemistry Research* **2013,** 52, (13), 4967-4972.
7. Wei, J.; Li, Z.; Gu, C.; Pan, Y.; Xing, N.-N.; Tong, J.; Guan, W., Determination of vaporization enthalpy for ionic liquids C(n)mim Lact (n=2, 3, 5) and applications of the molar surface Gibbs free energy. *Journal of Thermal Analysis and Calorimetry* **2016,** 125, (1), 547-556.
8. Wei, J.; Ma, T.; Ma, X.; Guan, W.; Liu, Q.; Yang, J., Study on thermodynamic properties and estimation of polarity of ionic liquids { C(n)mmim NTf2 (n=2, 4)}. *Rsc Advances* **2014,** 4, (58), 30725-30732.
9. Wei, J.; Fan, B.-H.; Pan, Y.; Xing, N.-N.; Men, S.-Q.; Tong, J.; Guan, W., Vaporization enthalpy and the molar surface Gibbs free energy for ionic liquids C(n)Dmim NTF2 (n=2, 4). *Journal of Chemical Thermodynamics* **2016,** 101, 278-284.
10. Luo, H.; Baker, G. A.; Dai, S., Isothermogravimetric determination of the enthalpies of vaporization of 1-alkyl-3-methylimidazolium ionic liquids. *The Journal of Physical Chemistry B* **2008,** 112, (33), 10077-10081.
11. Verevkin, S. P., Predicting enthalpy of vaporization of ionic liquids: A simple rule for a complex property. *Angewandte Chemie-International Edition* **2008,** 47, (27), 5071-5074.
12. Hong, M.; Sun, A.; Yang, Q.; Guan, W.; Tong, J.; Yang, J.-Z., Studies on properties of ionic liquids 1-alkyl-3-methylimidazolium lactate at temperatures from (288.15 to 333.15) K. *Journal of Chemical Thermodynamics* **2013,** 67, 91-98.
13. Tong, J.; Hong, M.; Chen, Y.; Wang, H.; Guan, W.; Yang, J.-Z., The surface tension, density and refractive index of amino acid ionic liquids: C(3)mim Gly and C(4)mim Gly. *Journal of Chemical Thermodynamics* **2012,** 54, 352-357.
14. Fang, D.-W.; Tong, J.; Guan, W.; Wang, H.; Yang, J.-Z., Predicting Properties of Amino Acid Ionic Liquid Homologue of 1-Alkyl-3-methylimidazolium Glycine. *Journal of Physical Chemistry B* **2010,** 114, (43), 13808-13814.
15. Hong, M.; Sun, A.; Liu, C.; Guan, W.; Tong, J.; Yang, J.-Z., Physico-chemical Properties of 1-Alkyl-3-methylimidazolium Propionate Ionic Liquids { C(n)mim Pro (n=3, 4, 5, 6)} from 288.15 K to 328.15 K. *Industrial & Engineering Chemistry Research* **2013,** 52, (44), 15679-15683.
16. Wei, J.; Chang, C.; Zhang, Y.; Hou, S.; Fang, D.; Guan, W., Prediction of thermophysical properties of novel ionic liquids based on serine C(n)mim Ser (n=3,4) using semiempirical

methods. *Journal of Chemical Thermodynamics* **2015,** 90, 310-316.

17. Clara, R. A.; Marigliano, A. C. G.; Solimo, H. N., Density, viscosity, isothermal (vapour plus liquid) equilibrium, excess molar volume, viscosity deviation, and their correlations for chloroform plus methyl isobutyl ketone binary system. *Journal of Chemical Thermodynamics* **2007,** 39, (2), 261-267.
18. Dragoescu, D., Refractive indices and their related properties for several binary mixtures containing cyclic ketones and chloroalkanes. *Journal of Molecular Liquids* **2015,** 209, 713-722.
19. Fenclová, D.; Vrbka, P.; Dohnal, V. r.; Řehák, K.; García-Miaja, G., (Vapour + liquid) equilibria and excess molar enthalpies for mixtures with strong complex formation. Trichloromethane or 1-bromo-1-chloro-2,2,2-trifluoroethane (halothane) with tetrahydropyran or piperidine. *The Journal of Chemical Thermodynamics* **2002,** 34, (3), 361-376.
20. Helm, R. V.; Lanum, W. J.; Cook, G. L.; Ball, J. S., Purification and Properties of Pyrrole, Pyrrolidine, Pyridine and 2-Methylpyridine. *The Journal of Physical Chemistry* **1958,** 62, (7), 858-862.
21. Lagemann, R. T.; McMillan, D. R.; Woolf, W. E., Temperature Variation of Ultrasonic Velocity in Liquids. *The Journal of Chemical Physics* **1949,** 17, (4), 369-373.
22. Colnay, M. E.; Vasseur, A.; Guerin, M., THE INFLUENCE OF NON-POLAR SOLVENTS ON MOLECULAR DIELECTRIC POLARIZATION .1. AN ATTEMPT TO SELECT BY EXPERIMENT APPROPRIATE EXPRESSIONS. *Journal of Chemical Research-S* **1983**, (9), 220-221.
23. Gill, D. S.; Singh, P.; Singh, J.; Singh, P.; Senanayake, G.; Hefter, G. T., ULTRASONIC VELOCITY, CONDUCTIVITY, VISCOSITY AND CALORIMETRIC STUDIES OF COPPER(I) AND SODIUM PERCHLORATES IN CYANOBENZENE, PYRIDINE AND CYANOMETHANE. *Journal of the Chemical Society-Faraday Transactions* **1995,** 91, (17), 2789-2795.
24. Sharma, B. R.; Singh, P. P., Excess Gibbs energies of mixing for some binary mixtures. *Journal of Chemical & Engineering Data* **1975,** 20, (4), 360-363.
25. Kyte, C.; Jeffery, G.; Vogel, A., 864. Physical properties and chemical constitution. Part XXVIII. Pyridine derivatives. *Journal of The Chemical Society (resumed)* **1960**.
26. Ortega, J., DENSITIES AND REFRACTIVE-INDEXES OF PURE ALCOHOLS AS A FUNCTION OF TEMPERATURE. *Journal of Chemical and Engineering Data* **1982,** 27, (3), 312-317.
27. Vijande, J.; Pineiro, M. M.; Garcia, J.; Valencia, J. L.; Legido, J. L., Density and surface tension variation with temperature for heptane+1-alkanol. *Journal of Chemical and Engineering Data* **2006,** 51, (5), 1778-1782.
28. Das, K. N.; Habibullah, M.; Rahman, I. M. M.; Hasegawa, H.; Uddin, M. A.; Saifuddin, K., Thermodynamic Properties of the Binary Mixture of Hexan-1-ol with m-Xylene at T = (303.15, 313.15, and 323.15) K. *Journal of Chemical & Engineering Data* **2009,** 54, (12), 3300-3302.
29. Neyband, R. S.; Yousefi, A.; Zarei, H., Experimental and Computational Thermodynamic Properties of (Benzyl Alcohol plus Alkanols) Mixtures. *Journal of Chemical and Engineering Data* **2015,** 60, (8), 2291-2300.

30. Venkatramana, L.; Sivakumar, K.; Gardas, R. L.; Reddy, K. D., Effect of chain length of alcohol on thermodynamic properties of their binary mixtures with benzylalcohol. *Thermochimica Acta* **2014,** 581, 123-132.
31. Weissler, A., Ultrasonic Investigation of Molecular Properties of Liquids. II.1 The Alcohols1a. *Journal of the American Chemical Society* **1948,** 70, (4), 1634-1640.
32. Huang, T.-T.; Yeh, C.-T.; Tu, C.-H., Densities, Viscosities, Refractive Indices, and Surface Tensions for the Ternary Mixtures of 2-Propanol + Benzyl Alcohol + 2-Phenylethanol at T = 308.15 K. *Journal of Chemical & Engineering Data* **2008,** 53, (5), 1203-1207.
33. Hiers, G. S.; Adams, R., OMEGA-CYCLOHEXYL DERIVATIVES OF VARIOUS NORMAL ALIPHATIC ACIDS. IV. *Journal of the American Chemical Society* **1926,** 48, (9), 2385-2393.
34. Hovorka, S.; Roux, A. H.; Roux-Desgranges, G.; Dohnal, V., Limiting partial molar excess heat capacities and volumes of selected organic compounds in water at 25 degrees C. *Journal of Solution Chemistry* **1999,** 28, (12), 1289-1305.
35. Shinomiya, T., DIELECTRIC-RELAXATION AND INTERMOLECULAR ASSOCIATION OF ALICYCLIC ALCOHOLS IN LIQUID AND SOLID STATES. *Bulletin of the Chemical Society of Japan* **1990,** 63, (4), 1087-1092.
36. Lasich, M.; Moodley, T.; Bhownath, R.; Naidoo, P.; Ramjugernath, D., Liquid–Liquid Equilibria of Methanol, Ethanol, and Propan-2-ol with Water and Dodecane. *Journal of Chemical & Engineering Data* **2011,** 56, (11), 4139-4146.
37. Zarei, H. A.; Shahvarpour, S., Volumetric Properties of Binary and Ternary Liquid Mixtures of 1-Propanol (1) + 2-Propanol (2) + Water (3) at Different Temperatures and Ambient Pressure (81.5 kPa). *Journal of Chemical & Engineering Data* **2008,** 53, (7), 1660-1668.
38. Ashcroft, S. J.; Clayton, A. D.; Shearn, R. B., Isothermal vapor-liquid equilibriums for the systems toluene-n-heptane, toluene-propan-2-ol, toluene-sulfolane, and propan-2-ol-sulfolane. *Journal of Chemical & Engineering Data* **1979,** 24, (3), 195-199.
39. Ritzoulis, G., EXCESS PROPERTIES OF THE BINARY-LIQUID SYSTEMS DIMETHYLSULFOXIDE + ISOPROPANOL AND PROPYLENE CARBONATE + ISOPROPANOL. *Canadian Journal of Chemistry-Revue Canadienne De Chimie* **1989,** 67, (6), 1105-1108.
40. Almasi, M., Densities and viscosities of binary mixtures of ethylmethylketone and 2-alkanols; application of the ERAS model and cubic EOS. *Thermochimica Acta* **2013,** 554, 25-31.
41. Ruostesuo, P.; Pirilahonkanen, P., THERMODYNAMIC AND SPECTROSCOPIC PROPERTIES OF 2-PYRROLIDINONES .2. DIELECTRIC-PROPERTIES OF 2-PYRROLIDINONE IN BINARY-MIXTURES. *Journal of Solution Chemistry* **1990,** 19, (5), 473-482.
42. Olivieri, G. V.; da Cunha, C. S.; Martins, L. d. S.; Paegle, P. A. M.; Nuncio, S. D.; Morandim-Giannetti, A. d. A.; Torres, R. B., Thermodynamic and spectroscopic study of binary mixtures of n-butylammonium oleate ionic liquid plus alcohol at T=288.15-308.15 K. *Journal of Thermal Analysis and Calorimetry* **2018,** 131, (3), 2925-2942.
43. Rafiee, H. R.; Frouzesh, F.; Miri, S., Volumetric properties for binary mixtures of ethyl acetate, vinyl acetate and tert-butyl acetate with 1-propanol and iso-butanol at T = (293.15-313.15) K and P=0.087 MPa. *Journal of Molecular Liquids* **2016,** 213, 255-267.

.

44. Zaoui-Djelloul-Daouadji, M.; Mokbel, I.; Bahadur, I.; Negadi, A.; Jose, J.; Ramjugernath, D.; Ebenso, E. E.; Negadi, L., Vapor-liquid equilibria, density and sound velocity measurements of (water or methanol or ethanol+1,3-propanediol) binary systems at different temperatures. *Thermochimica Acta* **2016,** 642, 111-123.
45. Vasanthakumar, A.; Bahadur, I.; Redhi, G. G.; Gengan, R. M.; Anand, K., Synthesis, characterization and thermophysical properties of ionic liquid N-methyl-N-(2 ',3 '-epoxypropyl)-2-oxopyrrolidinium chloride and its binary mixtures with water or ethanol at different temperatures. *Journal of Molecular Liquids* **2016,** 219, 685-693.
46. Yao, H.; Zhang, S.; Wang, J.; Zhou, Q.; Dong, H.; Zhang, X., Densities and Viscosities of the Binary Mixtures of 1-Ethyl-3-methylimidazolium Bis(trifluoromethylsulfonyl)imide with N-Methyl-2-pyrrolidone or Ethanol at T = (293.15 to 323.15) K. *Journal of Chemical and Engineering Data* **2012,** 57, (3), 875-881.
47. Yan, J.-H.; Dai, L.-Y.; Wang, X.-Z.; Chen, Y.-Q., Densities and Viscosities of Binary Mixtures of Cyclopropanecarboxylic Acid with Methanol, Ethanol, Propan-1-ol, and Butan-1-ol at Different Temperatures. *Journal of Chemical and Engineering Data* **2009,** 54, (3), 1147-1152.
48. Zarei, H. A.; Mirhidari, N.; Zangeneh, Z., Densities, Excess Molar Volumes, Viscosity, and Refractive Indices of Binary and Ternary Liquid Mixtures of Methanol (1) + Ethanol (2)+1,2-Propanediol (3) at P=81.5 kPa. *Journal of Chemical and Engineering Data* **2009,** 54, (3), 847-854.
49. Fan, W.; Zhou, Q.; Zhang, S.; Yan, R., Excess molar volume and viscosity deviation for the methanol plus methyl methacrylate binary system at T = (283.15 to 333.15) K. *Journal of Chemical and Engineering Data* **2008,** 53, (8), 1836-1840.
50. Tong, J.; Hong, M.; Chen, Y.; Wang, H.; Guan, W.; Yang, J.-Z., The surface tension, density and refractive index of amino acid ionic liquids: [C3mim][Gly] and [C4mim][Gly]. *The Journal of Chemical Thermodynamics* **2012,** 54, 352-357.
51. Lide, D. R., *CRC handbook of chemistry and physics*. CRC press: 2004; Vol. 85.
